# Stratification of ovarian cancer borderline from high-grade serous carcinoma patients by quantitative serum NMR spectroscopy of metabolites, lipoproteins, and inflammatory markers

**DOI:** 10.3389/fmolb.2023.1158330

**Published:** 2023-04-19

**Authors:** Gyuntae Bae, Georgy Berezhnoy, André Koch, Claire Cannet, Hartmut Schäfer, Stefan Kommoss, Sara Brucker, Nicolas Beziere, Christoph Trautwein

**Affiliations:** ^1^ Werner Siemens Imaging Center, Department of Preclinical Imaging and Radiopharmacy, University Hospital Tübingen, Tübingen, Germany; ^2^ Department of Women’s Health, University Hospital Tübingen, Tübingen, Germany; ^3^ Bruker BioSpin GmbH, Ettlingen, Germany; ^4^ Cluster of Excellence CMFI (EXC 2124) “Controlling Microbes to Fight Infections”, Eberhard Karls University of Tübingen, Tübingen, Germany

**Keywords:** metabolomics, tumor progression, metastasis, glycoprotein, CA-125, biomarker, diagnostics, precision medicine

## Abstract

**Background:** Traditional diagnosis is based on histology or clinical-stage classification which provides no information on tumor metabolism and inflammation, which, however, are both hallmarks of cancer and are directly associated with prognosis and severity. This project was an exploratory approach to profile metabolites, lipoproteins, and inflammation parameters (glycoprotein A and glycoprotein B) of borderline ovarian tumor (BOT) and high-grade serous ovarian cancer (HGSOC) for identifying additional useful serum markers and stratifying ovarian cancer patients in the future.

**Methods:** This project included 201 serum samples of which 50 were received from BOT and 151 from high-grade serous ovarian cancer (HGSOC), respectively. All the serum samples were validated and phenotyped by ^1^H-NMR-based metabolomics with *in vitro* diagnostics research (IVDr) standard operating procedures generating quantitative data on 38 metabolites, 112 lipoprotein parameters, and 5 inflammation markers. Uni- and multivariate statistics were applied to identify NMR-based alterations. Moreover, biomarker analysis was carried out with all NMR parameters and CA-125.

**Results:** Ketone bodies, glutamate, 2-hydroxybutyrate, glucose, glycerol, and phenylalanine levels were significantly higher in HGSOC, while the same tumors showed significantly lower levels of alanine and histidine. Furthermore, alanine and histidine and formic acid decreased and increased, respectively, over the clinical stages. Inflammatory markers glycoproteins A and B (GlycA and GlycB) increased significantly over the clinical stages and were higher in HGSOC, alongside significant changes in lipoproteins. Lipoprotein subfractions of VLDLs, IDLs, and LDLs increased significantly in HGSOC and over the clinical stages, while total plasma apolipoprotein A1 and A2 and a subfraction of HDLs decreased significantly over the clinical stages. Additionally, LDL triglycerides significantly increased in advanced ovarian cancer. In biomarker analysis, glycoprotein inflammation biomarkers behaved in the same way as the established clinical biomarker CA-125. Moreover, CA-125/GlycA, CA-125/GlycB, and CA-125/Glycs are potential biomarkers for diagnosis, prognosis, and treatment response of epithelial ovarian cancer (EOC). Last, the quantitative inflammatory parameters clearly displayed unique patterns of metabolites, lipoproteins, and CA-125 in BOT and HGSOC with clinical stages I–IV.

**Conclusion:** ^1^H-NMR-based metabolomics with commercial IVDr assays could detect and identify altered metabolites and lipoproteins relevant to EOC development and progression and show that inflammation (based on glycoproteins) increased along with malignancy. As inflammation is a hallmark of cancer, glycoproteins, thereof, are promising future serum biomarkers for the diagnosis, prognosis, and treatment response of EOC. This was supported by the definition and stratification of three different inflammatory serum classes which characterize specific alternations in metabolites, lipoproteins, and CA-125, implicating that future diagnosis could be refined not only by diagnosed histology and/or clinical stages but also by glycoprotein classes.

## 1 Introduction

Ovarian cancer (OC) has been considered highly life-threatening (Clarke-Pearson, 2009), and worldwide, OC incidents and deaths are 88.01% and 84.20%, respectively (Zhou et al., 2021). To date, more than 30 different histology types of OC have been described, and epithelial OC (EOC) that starts to proliferate in the epithelial layer covering the ovary is the most common and accounts for more than 95% of OC malignancy (Desai et al., 2014; Kaku et al., 2003). Furthermore, EOC is classified into five subtypes, of which high-grade serous ovarian cancer (HGSOC) is the most frequently diagnosed (Prat and FIGO Committee on Gynecologic Oncology, 2014).

OC relies on a variety of energy metabolites to develop; in particular, OC has high propensity on Warburg and reverse Warburg effects (Schwartz et al., 2017; Li et al., 2019; Wang and Li, 2020). As *Otto Warburg* demonstrated, neoplasms showed highly increased metabolic rates that were characterized by a high uptake of glucose as a primary energy source and the production of an excessive amount of lactate even in the presence of oxygen (Warburg et al., 1927). This process is called the Warburg effect, involving the alteration of metabolic enzymes such as hexokinase 2 (HK2) (Wang et al., 2014), pyruvate kinase type M2 (PKM2) (Wong et al., 2015), glucose transporter 1 (GLUT1) (Mayer et al., 2014), lactate dehydrogenase (LDH), and lactate transporter [monocarboxilate transporter (MCTs)] (Fantin et al., 2006). On the other hand, the reverse Warburg effect reflects that adjacent cancer cells are metabolically supported by cancer-associated fibroblasts (CAFs), which can undergo HIF-1α-induced autophagosomal degradation and aerobic glycolysis. Following this, lactate, 3-hydroxybutyrate, and glutamines are released into the tumor microenvironment (TME). In turn, the cancer cells utilize lactate and 3-hydroxybutyrate and glutamine for adenosine triphosphate (ATP) and glutathione production, respectively (Fu et al., 2017; Thuwajit et al., 2018; Wilson et al., 2019). Furthermore, OC patients end up with cachexia, anorexia, and death due to increased resting metabolism alongside peritoneal metastasis and progression (Archid et al., 2019; Hilal et al., 2017).

In addition to energy production by polar metabolites, cancer cells also utilize lipids to survive and proliferate (Butler et al., 2020). The consequence of altered lipid metabolic pathways, increased *de novo* lipogenesis and lipolysis *via* exogenous (dietary) and endogenous uptakes, respectively, allows cancer cells to enhance membrane biogenesis and ATP production (Butler et al., 2020) and then evades apoptosis (Swinnen et al., 2006; Menendez and Lupu, 2007; DeBerardinis et al., 2008). The two major sources to obtain such supplies endogenously are the omentum majus adipocytes, especially in OC (Nieman et al., 2011), and lipoproteins that are mainly synthesized by the liver carrying cholesterols (CL) and triglycerides (TG) to cancer cells (Brown, 2007; Maran et al., 2021). Moreover, inflammation is related to EOC initiation and progression. Some sources of inflammation are retrograde menstruation, obesity, ovulation, polycystic ovary syndrome (PCOS), talc exposure, infections (Savant et al., 2018), postmenopausal event (Jia et al., 2018), and dysbiotic microbiome (Wang et al., 2020). As a result, systemic inflammation occurs alongside changes in lipoproteins, which promotes carcinogenesis and malignant metastasis (Greten and Grivennikov, 2019; Georgila et al., 2019).

Detection of OC at an early stage (clinical stage I or II) is a crucial step for curing OC. Approximately, the chance to diagnose OC at the early stage is about 20%, and it allows to increase the 5- and 10-year overall survival of the patients by 71.4% and 53%, respectively (Kim et al., 2018; Peres et al., 2019). However, to date, an early-stage diagnosis is hard to achieve due to an unclear understanding of OC development and tumor pathogenesis (Bast et al., 2009; Bowtell et al., 2015).

Until now, in addition to conventional strategies to determine OC development, there is no specific way to diagnose and detect OC at the early stage among women who are exposed to inevitable risks, such as aging (Setiawan et al., 2012) and menopausal status (Nichols et al., 2006). A conventional diagnostic approach is blood test of the cancer antigen marker CA-125 (Gupta and Lis, 2009) and transvaginal ultrasound (van Nagell and Hoff, 2013). Yet, each diagnostic test has a drawback; CA-125 is influenced by a number of OC-unrelated conditions (Kobayashi et al., 2012), and transvaginal ultrasound cannot distinguish between benign tumor and cancer (van Nagell and Hoff, 2013), thus providing a low specification. Moreover, other imaging approaches, including computed tomography (Iyer and Lee, 2010), magnetic resonance imaging (Prayer et al., 1993; Low et al., 1995), and positron emission tomography/computed tomography (Yamamoto et al., 2008; Karantanis et al., 2012), are not sensitive to diagnose ovarian tumor and cancer. In other words, morphological changes and biological properties are not enough to evaluate the disease progression in OC. Hence, discovering additional biomarkers is, indeed, one of the clinical needs.

In this project, metabolite and lipoprotein profiles of borderline ovarian tumor and HGSOC patients’ serum were investigated alongside inflammatory markers by commercially available quantitative IVDr NMR standard operating procedures (SOPs) as provided by Bruker BioSpin. Uni- and multivariate statistics were applied to identify NMR-based alterations based on patients’ diagnosed histology and clinical stage. The correlation of glycoproteins and OC cancer antigen markers [CA-125, carcinoembryonic antigen (CEA), and carbohydrate antigen 19-9 (CA 19-9)] was studied for the first time.

## 2 Materials and methods

### 2.1 Patients’ clinical information and sample collection and storage

**FIGURE 1:**
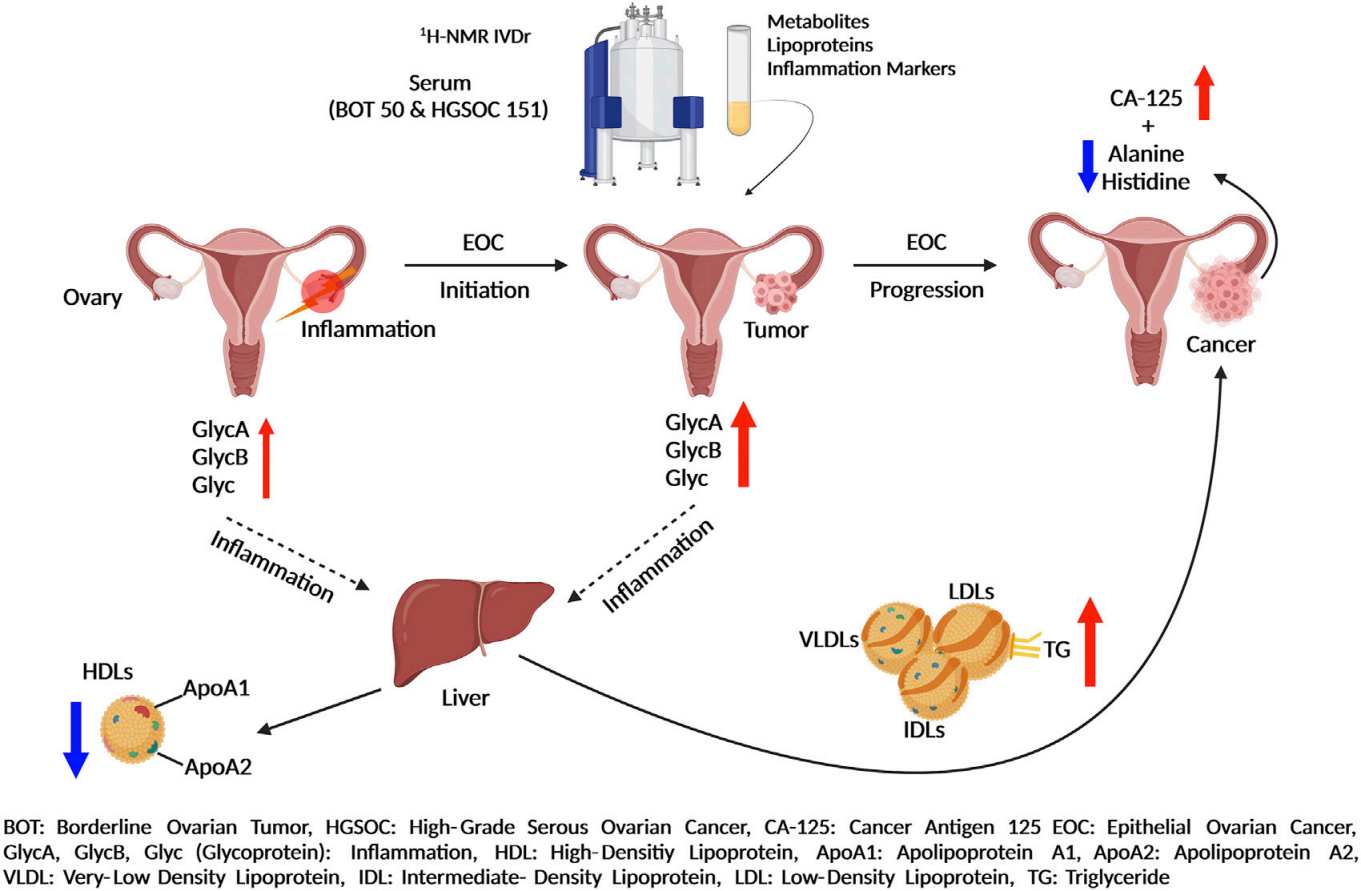
Summary of epithelial ovarian cancer development and progression by *in vitro* diagnostics research ^1^H-NMR-based metabolomics assays.

Table 1 describes clinical and pathological characteristics of the patients. A total of 201 serum samples in 2 mL aliquots (50 of BOT and 151 of HGSOC) with patients’ information were provided by the biobank (freezer at −80°C) of Women’s Health at Universitätsklinikum Tübingen. All patients gave written consent, and samples were collected under the ethical approval number 208/2021BO2. A graphical summary of the key findings of this study is provided within Figure 1.

**TABLE 1:** Summery of patient characteristics.

Number of patients	201
Age (mean, minimum, and maximum)	58.75 (18–87)
Gender	Female
Cancer type	Ovarian cancer
Histology	
High-grade serous ovarian cancer	151 (75%)
Endometrioid borderline tumor	2 (1%)
Mucinous borderline tumor	16 (8%)
Serous borderline tumor	29 (14%)
N/A but diagnosed as borderline tumor	3 (2%)
Tumor grading	
GB	50 (25%)
G3	151 (75%)
FIGO stage	
I	43 (21%)
II	13 (6.5%)
III	81 (40%)
III-IV	1 (0.5%)
IV	33 (16%)
N/A	30 (15%)
Treatment status	
Pre-treated	25 (12%)
Untreated	150 (75%)
N/A	26 (13%)

N/A: not applicable; FIGO: International Federation of Gynecology and Obstetrics.

### 2.2 ^1^H-NMR spectroscopy equipment and spectra acquisition

**FIGURE 2:**
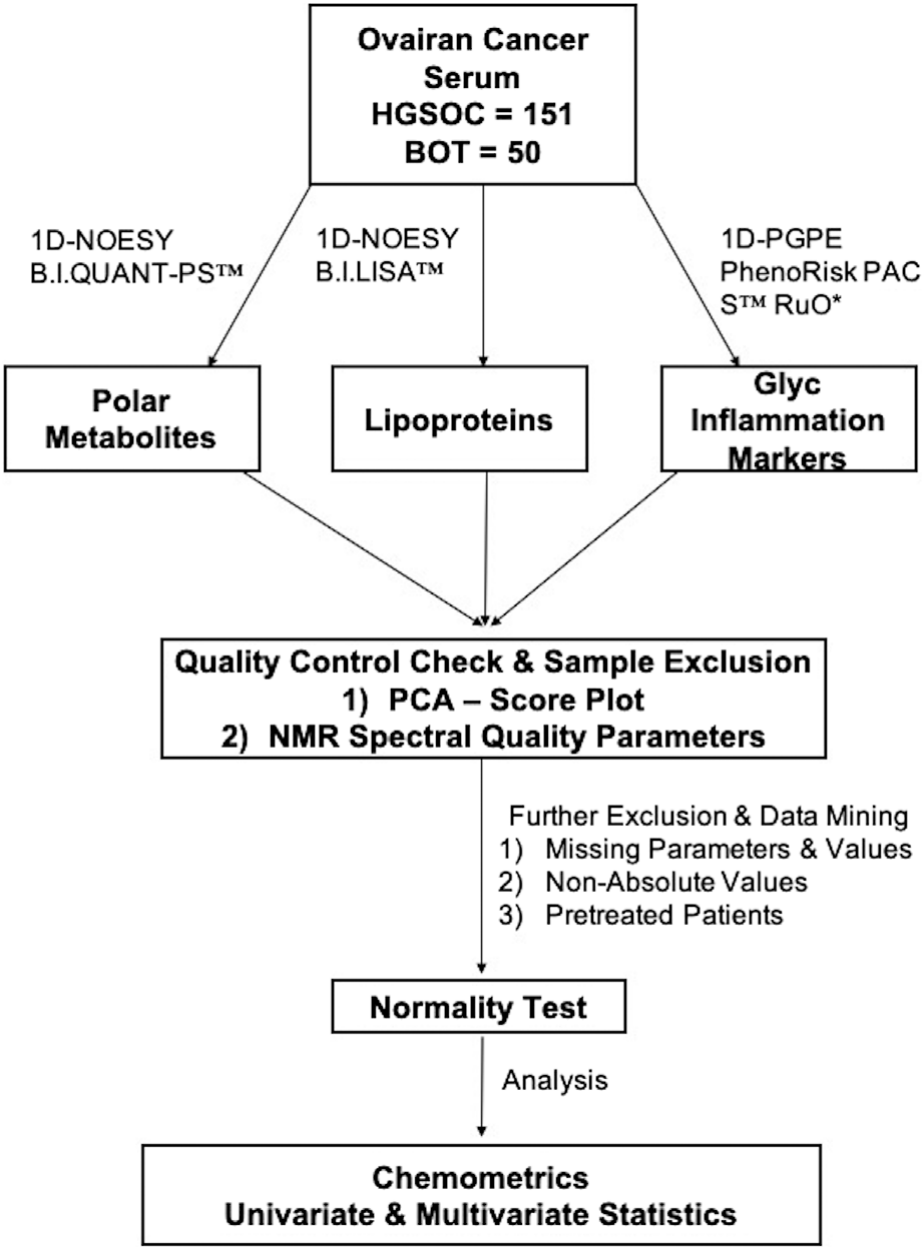
Metabolomics workflow. An overview of the data workflow in metabolomics for the identification of NMR-based alternations from borderline ovarian tumor and high-grade serous ovarian cancer serum samples.

^1^H-NMR spectroscopy (Bruker Avance III HD 14.10 T) was operated at 600 MHz with a triple-resonance (TXI) room temperature 5 mm probe at 310 K. All samples were measured, quantified and analyzed in a similar scheme (Figure 2).

### 2.3 Sample preparation for Bruker Avance IVDr NMR analysis

The serum was thawed at room temperature for 30 min. Following this, the serum samples were then placed inside a box with ice to prevent degradation. The next steps were performed according to the Bruker IVDr NMR SOP in brief by adding 400 μL of Bruker Plasma Buffer and 400 µL of the serum into a 1.5 mL Eppendorf tube and then transferring 600 μL of the solution into a 5 mm NMR tube for measurement.

### 2.4 Quantification of the measured serum and evaluation of quality control

All the serums were measured with a nuclear Overhauser spectroscopy experiment (1D-NOESY) for 4 min to quantify polar 40 metabolites and 112 lipoproteins by small-molecule metabolites (B.I.QUANT-PS™) and Bruker IVDr Lipoprotein Subclass Analysis (B.I.LISA™), respectively (Bruker.com, 2022a). The inflammatory analytes GlycA, GlycB, and Glyc (addition of GlycA and GlycB) were measured with a sequence of pulse gradient perfect echo experiment (1D-PGPE) and quantified by PhenoRisk PACS™ RuO* (Bruker.com, 2022b). Each serum was subject to a quality control test by B.I. methods (Bruker.com, 2022b).

### 2.5 Chemometrics

This is an exploratory study without prior sample size calculation. Statistical analysis was performed using the MetaboAnalyst 5.0 toolbox (Xia et al., 2009). The quantified analytes were normalized to the sample volume. The missing values of metabolites were replaced by LoDs (1/5 of the minimum positive value of each variable), and the missing values of lipoproteins were estimated by k-nearest neighbors (KNN) feature-wise. Additionally, the estimation of the missing value of metabolites and lipoproteins was carried out using the KNN for correlation between metabolites, lipoproteins, inflammation, and CA-125 markers. Serum samples that appeared as outliers by principal component analysis (PCA) and failed to pass an NMR experiment quality test were excluded. Of note, all pre-treated patients (radiotherapy and/or chemotherapy) were excluded from statistical analysis. Moreover, all patients with missing and non-absolute levels of cancer antigen markers, such as CA-125, CEA, and CA 19-9, were excluded from comparative and correlation analysis.

### 2.6 Comparative statistics

It was performed using Prism software 9. Normally distributed data were subject to an unpaired t-test and ordinary ANOVA tests after the F-test. Skewed data were statistically analyzed with Mann–Whitney and Kruskal–Wallis tests. A value of *p* < 0.05 was considered significant. Of note, a false discovery rate (FDR) was applied to correct the *p*-value.

### 2.7 Univariate and multivariate analyses

A volcano plot was used only for two group-based comparisons, to analyze altered metabolites and lipoproteins. A value of *p* < 0.05 and fold change (FC) cutoff >1.2 were considered statistically and biologically significant. In multivariate analysis, a PCA score plot, PCA biplot, and sparse partial least square discriminant analysis (sPLSDA) score plot were used to observe the clusters and separation based on the respective comparison. Correlation analysis is independent of the group. The data were log-transformed, pareto-scaled, and then, analyzed by Spearman’s correlation with the PatternHunter tool of MetaboAnlyst 5.0 for skewed data. Moreover, correlation analysis was performed to observe the correlation between Glyc NMR parameters and cancer antigen markers (CEA and CA 19-9), the data of which were log-transformed. Last, a k-means clustering plot was performed based on the quantitative inflammatory parameters (GlycA, GlycB, and Glyc), and then, we further analyzed the NMR-based alternation of metabolites and lipoproteins with the CA-125 marker by the sPLSDA score plot and comparative statistics. Of note, all of these parameters were also log-transformed and pareto-scaled.

### 2.8 Biomarker analysis

Inflammation markers (GlycA, GlycB, and Glyc) were subject to a comparative statistical analysis and classical univariate receiver operating characteristic (ROC) curve analysis, to observe how accurate these markers are in distinguishing OC patients. Furthermore, all NMR parameters and CA-125 were log-transformed and pareto-scaled, and biomarker analysis was carried out based on the principle “compute and include metabolite ratios.”

## 3 Results

### 3.1 Polar metabolites and lipoproteins vary in histology of ovarian cancer with clinical stages I–IV

**FIGURE 3:**
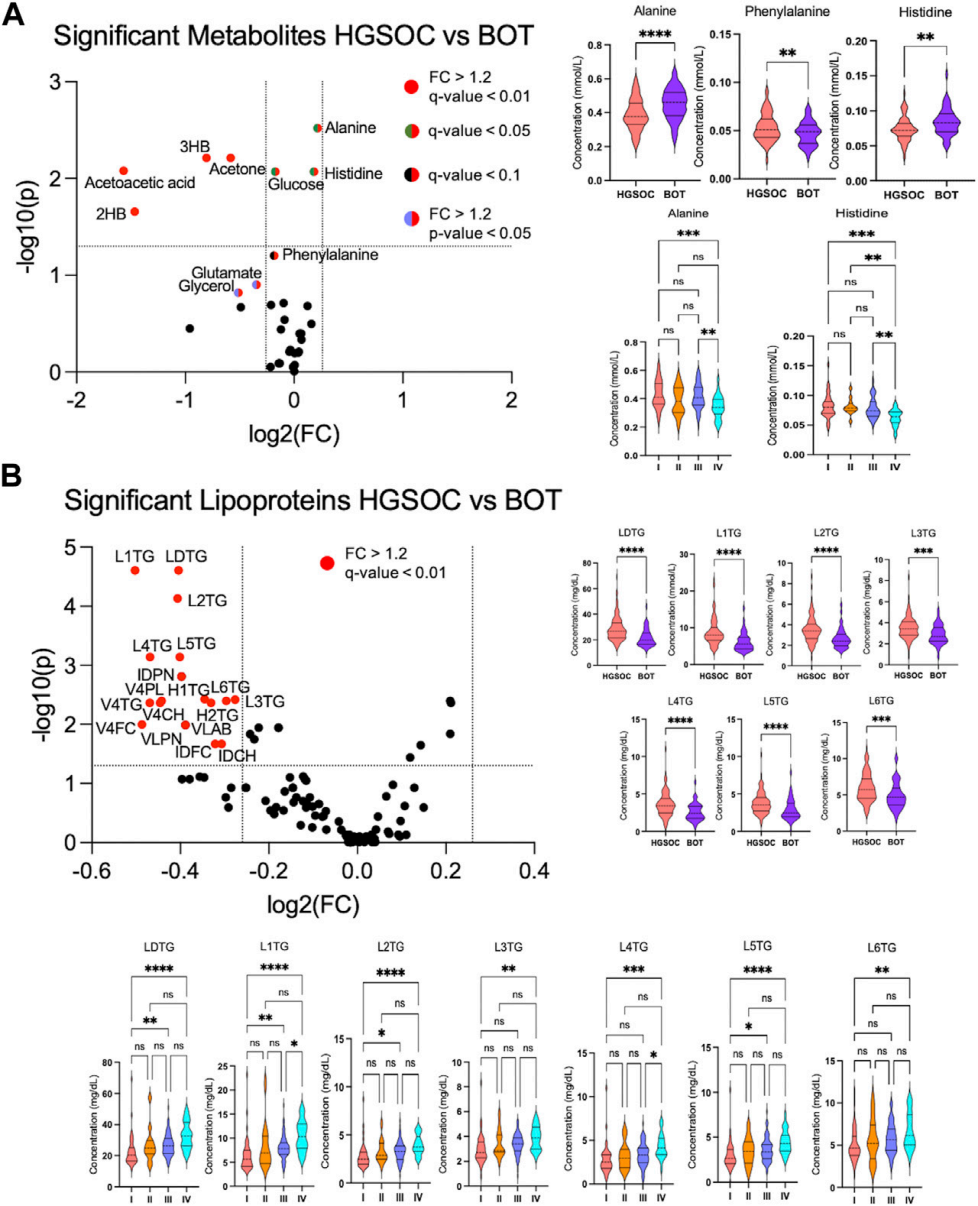
Altered metabolites and lipoproteins in borderline ovarian tumor (BOT) and high-grade serous ovarian cancer (HGSOC) with clinical stages I–IV. From left: **(A, B)** volcano plot showing statistically and biologically significant metabolites and lipoproteins in the histology of ovarian cancer; red plots indicate upregulation in high-grade serous ovarian cancer (fold change >1.2 and *p*-value <0.05). From right: **(A, B)** violin plots by comparative statistics showing significantly altered alanine (FDR <0.01), phenylalanine (FDR <0.1), and histidine (FDR <0.01) in high-grade serous ovarian cancer (** <0.01 and ****<0.0001) and significantly altered alanine and histidine over the clinical stages (q-value = ** <0.01 and ***< 0.001). From right: (B and bottom) violin plots displaying significantly altered lipoproteins in high-grade serous ovarian cancer (FDR <0.01, ***< 0.001, and ****<0.0001) and over the clinical stages (q-value = (*<0.05, ** <0.01, ***< 0.001, and ****<0.0001).

Volcano analysis and comparative statistics were carried out. In the volcano plot, ketone bodies, glutamate, and glycerol were upregulated in HGSOC compared to BOT (Figure 3A). The rest of the metabolites were found significant by comparative statistics; alanine and histidine were significantly higher in BOT (Figure 3A), and glucose, 2-hydroxybutyric acid (Supplementary Figure S1), and phenylalanine (Figure 3A) were significantly higher in HGSOC. A multivariate analysis was further performed to observe any discernible patterns in the metabolite profiles of BOT and HGSOC. HGSOCs were closely clustered to BOT (Supplementary Figure S9A), yet they tended to be separate from BOTs, which was due to glucose and lactic acid relevant to OC development.

In terms of the clinical stages, acetoacetic acid, formic acid, and histidine were significantly different between OC with stages I–IV. Acetoacetic acid was observed to be significantly lower in OC with stage I than in OC with stages II and IV (Supplementary Figure S1), while alanine was significantly higher in OC with stages I and III than OC with stage IV (Figure 3A). Formic acid (Supplementary Figure S1) and histidine (Figure 3A) increased and decreased over the clinical stage, respectively.

From the quantitative lipoprotein panel, the parameters L1TG, LDTG, L2TG, L4TG, L5TG, IDAB, IDPN, H2TG, L3TG, L3TG, V4PL, V4CH, H1TG, V4TG, V4FC, VLAB, VLPN, IDCH, and IDFC were upregulated in HGSOC compared to BOT (Figure 3B). It can be estimated that these increased lipoproteins carry TG, phospholipids, CL, and free CL to the OC, and at the same time, TG are transported back to the liver by H1TG and H2TG. Moreover, total TG (TPTG), total cholesterols (TPCH), high-density lipoprotein cholesterol (HDCH), and low-density lipoprotein cholesterol (LDCH) were not significant between HGSOC and BOT (Supplementary Figure S2). The multivariate analysis showed that HGSOC and BOT were clustered next to each other, and the separation was driven by TBPN and LDPN (Supplementary Figures S9C, S9D). Indeed, lipoproteins seemed to facilitate OC development.

In the clinical stage-based comparison, H3FC, H4A1, H4A2, H4FC, HDA1, HDCH, HDTG, TPA1, TPA2, V5TG, and ABA1 showed significant changes, while the rest of the lipoproteins were observed the same way as in the histology-based comparison (Supplementary Figure S3). High-density lipoproteins (HDLs) apolipoprotein A-1 (ApoA1) and apolipoprotein A-2 (ApoA2), and low-density lipoproteins (LDLs), very-low-density lipoproteins (VLDLs), and intermediate-density lipoproteins (IDLs) tended to decrease and increase, respectively, over the clinical stage. Moreover, H1TG, H2TG, and HDTG increased over the clinical stage (Supplementary Figure S3).

### 3.2 Glycoprotein inflammation markers of borderline ovarian tumor and high-grade serous ovarian cancer stages I–IV vary according to each other

**FIGURE 4:**
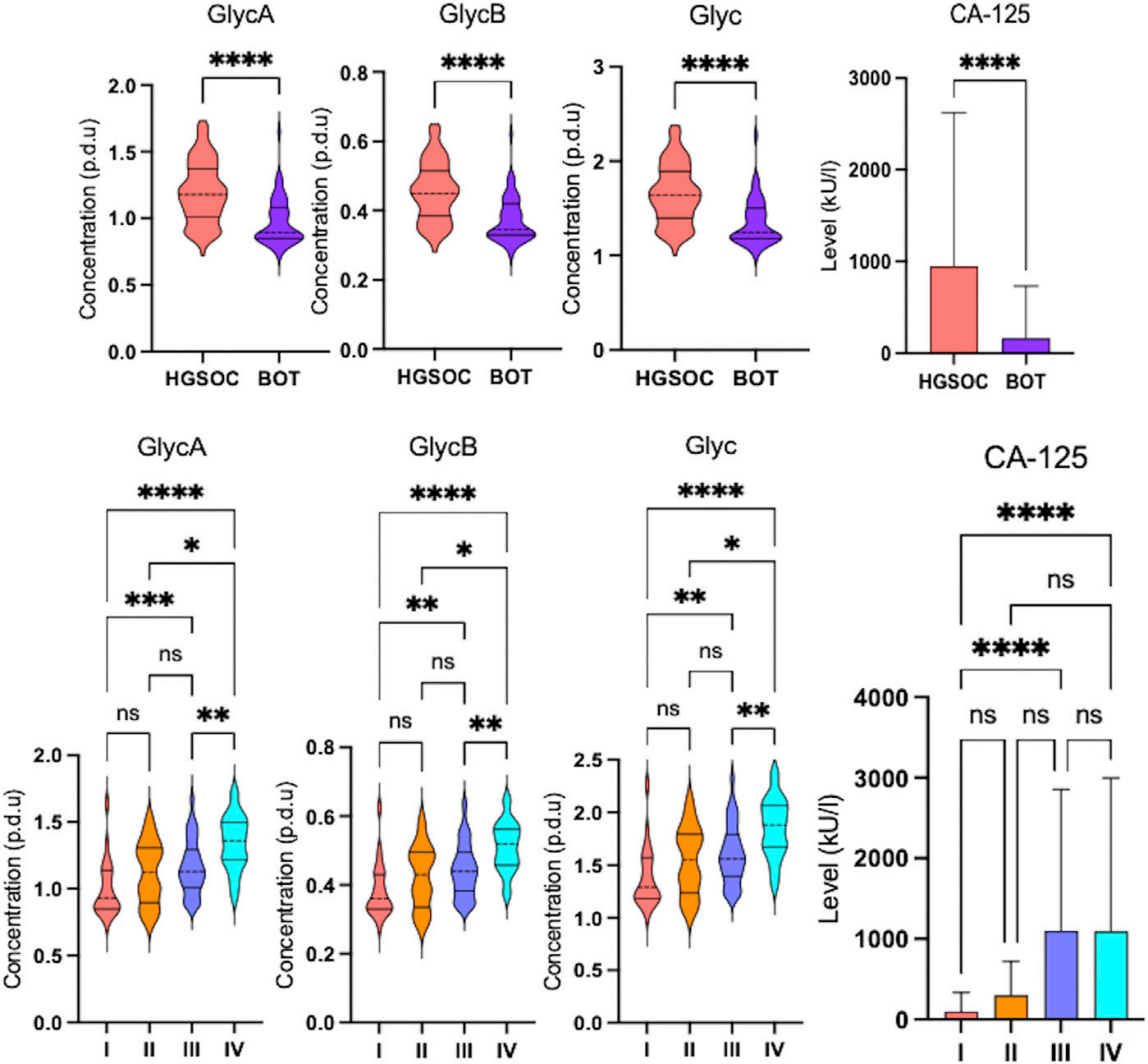
Altered glycoprotein inflammation and cancer antigen-125 markers in ovarian cancer serum samples. Violin and box plots showing significant increase in glycoprotein inflammation and cancer antigen-125 markers over the clinical stages (q-value = *<0.05, ** <0.01, ***< 0.001, and ****<0.0001) that they are higher in high-grade serous ovarian cancer than in borderline ovarian tumor (FDR <0.001, *<0.05, ** <0.01, ***< 0.001, and ****<0.0001).

Inflammation markers such as glycoprotein A (GlycA), glycoprotein B (GlycB), and overall Glyc were significantly higher in HGSOC than in BOT (Figure 4), indicating that inflammation occurred during OC development. The inflammation based on Glyc results also increased over the clinical stages where significance was observed between stages I vs. IV, I vs. III, II vs. IV, and III vs. IV (Figure 4). The multivariate analysis clearly showed that glycoprotein-assessed inflammation varied between the diagnosed histology, and the altered inflammation was indeed related to their tumor progression (Supplementary Figures S9E, S9F).

### 3.3 Glycoprotein inflammation markers predict effectiveness of the treatment and are promising add-ons for diagnosis and prognosis of ovarian cancer

**FIGURE 5:**
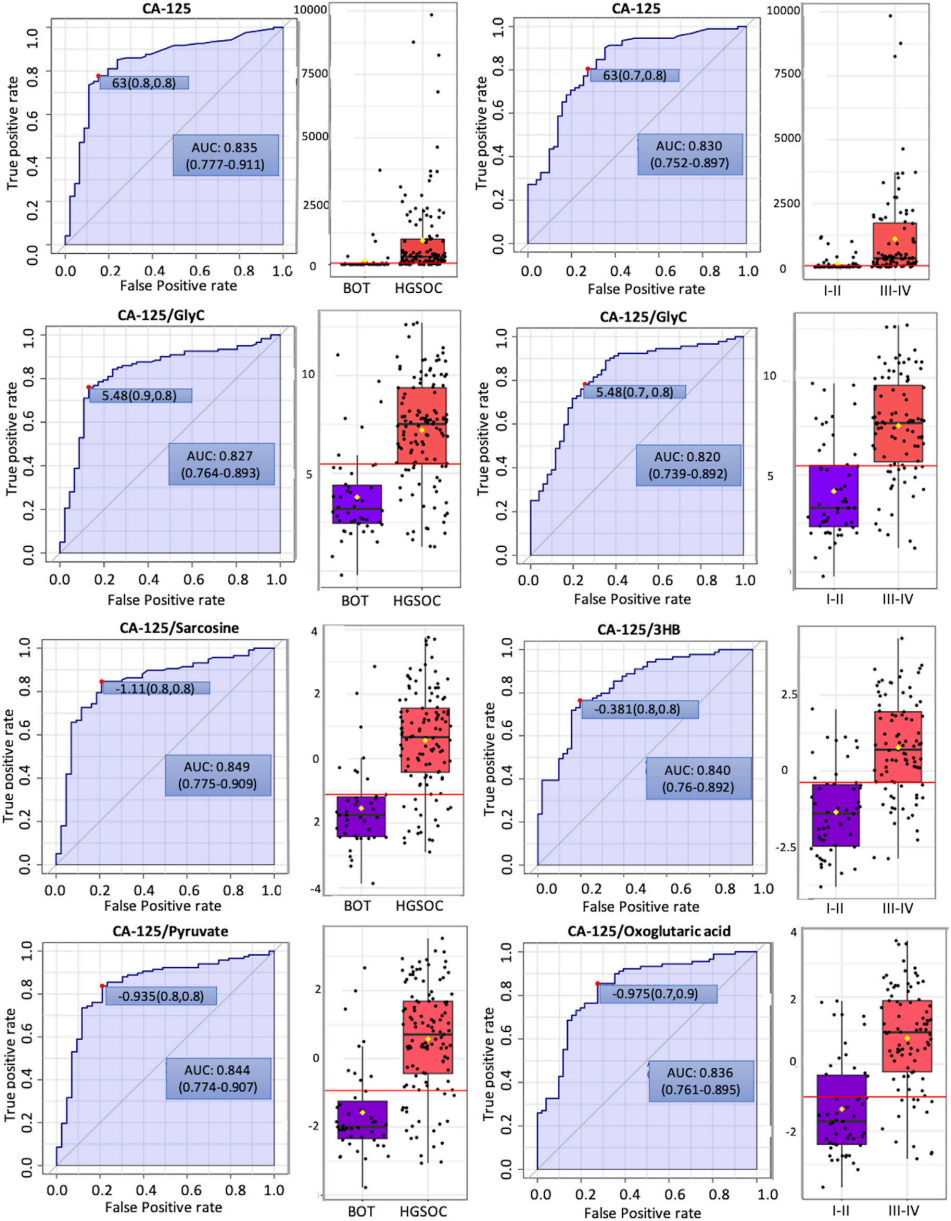
Potential biomarker candidates to cancer antigen-125 markers for ovarian cancer diagnosis and prognosis. The optimal cutoff was based on the closest to the top left corner principle and is indicated by the red dot in all the ROC curves. Black dots and yellow diamond represent the level of cancer antigen-125 and each ratio and mean concentration of cancer antigen-125 and each ratio, respectively.

In order to see whether GlycA, GlycB, and the sum of Glyc possess potential for diagnosis and prognosis, comparative statistical analysis was carried out in a treatment-based comparison. The inflammation markers were not significant between the treatment statuses (Supplementary Figure S4). No significant change was further confirmed by cross-validation with the “leave one out cross-validation” method (LOOCV); Q2 was negative (Supplementary Table S16), which means that the group was not predictive at all, and PLS-DA (partial least square discriminant analysis) would not provide important information (Szymańska et al., 2012). Additionally, the cancer antigen marker CA-125 that is used to investigate the effectiveness of radiotherapy (Aliomar et al., 2013) and chemotherapy (Wang et al., 2019) was subject to comparative statistics. Hereby, the result shows non-significance between the overall groups (Supplementary Figure S4), yet it was significant in comparing BOT vs. HGSOC and clinical stages (Figure 4). Biomarker analysis shows that the inflammation markers were able to distinguish between BOT vs. HGSOC (Supplementary Figure S7) and I–II vs. III–IV (Supplementary Figure S5), as CA-125. In this study, all NMR parameters alone were not good enough to classify both histology and clinical stage of OC (Supplementary Tables S24, S25). However, we could see that the area under the curve (AUC) of CA-125/sarcosine, CA-125/pyruvate, CA-125/3HB, and CA-125/oxoglutaric acid was higher than that of CA-125 (Figure 5). Histology of OC was classified by CA-125/sarcosine and CA-125/pyruvate, while the classification of the clinical stage of OC was achieved by CA-125/3HB and CA-125/oxoglutaric acid. Moreover, increased ratio values of CA-125/GlycA (Supplementary Figure S6), CA-125/GlycB (Supplementary Figure S6), and CA-125/Glyc (Figure 5) within AUC analysis helped in classifying both the histology and clinical stage of OC.

### 3.4 Quantitative inflammatory parameters clearly characterize specific patterns of metabolites, lipoproteins, and CA-125 in ovarian tumor and cancer with clinical stages I–IV

**FIGURE 6:**
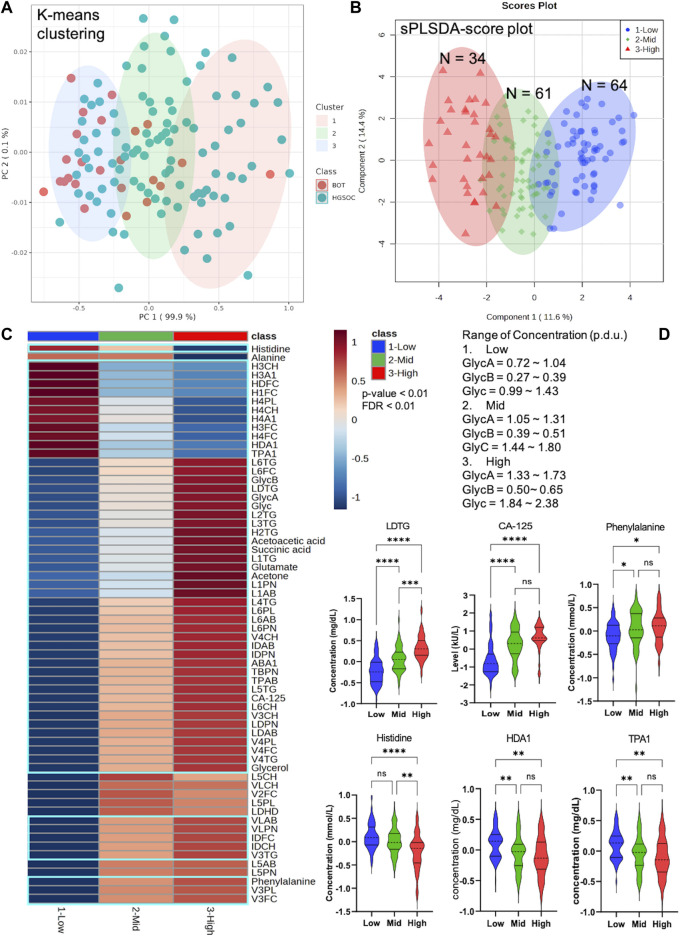
Unique pattern of metabolites, lipoproteins, and CA-125 in different Glyc classes that include borderline ovarian tumor (BOT) and high-grade serous ovarian cancer (HGSOC) with clinical stages. **(A)** K-means clustering based on NMR-based inflammatory concentration. **(B)** sPLSDA score plot with clear distribution of ovarian tumor and cancer at different inflammatory levels. **(C)** Heatmap displaying significantly altered metabolites, lipoproteins, and CA-125 of ovarian tumor and cancer at different inflammatory concentrations (*p*-value <0.01 and FDR <0.01). **(D)** Selected violin plots by comparative statistics showing significantly altered metabolites, lipoproteins, and CA-125 involved in inflammation (*q*-value = *<0.01, **<0.001, ***<0.0001, and ****<0.00001).

K-means clustering was performed with the quantitative inflammatory parameters, where we could distinguish different inflammatory classes. In other words, quantitative inflammatory parameters varied according to each cluster (Figure 6). We then carried out sPLSDA and comparative statistics to observe the NMR-based alternations and CA-125 based on the inflammatory classes. Each class was clearly separated along with specific and unique changes in metabolites, lipoproteins, and CA-125 (Figure 6). Moreover, the model was cross-validated with LOOCV; the error rate was 8.8% at component 1 (Supplementary Figure S10), indicating that different glycoprotein classes perform good classification.

### 3.5 Correlation of glycoprotein inflammation markers with the established cancer markers CA-125, CEA, and CA 19-9

**FIGURE 7:**
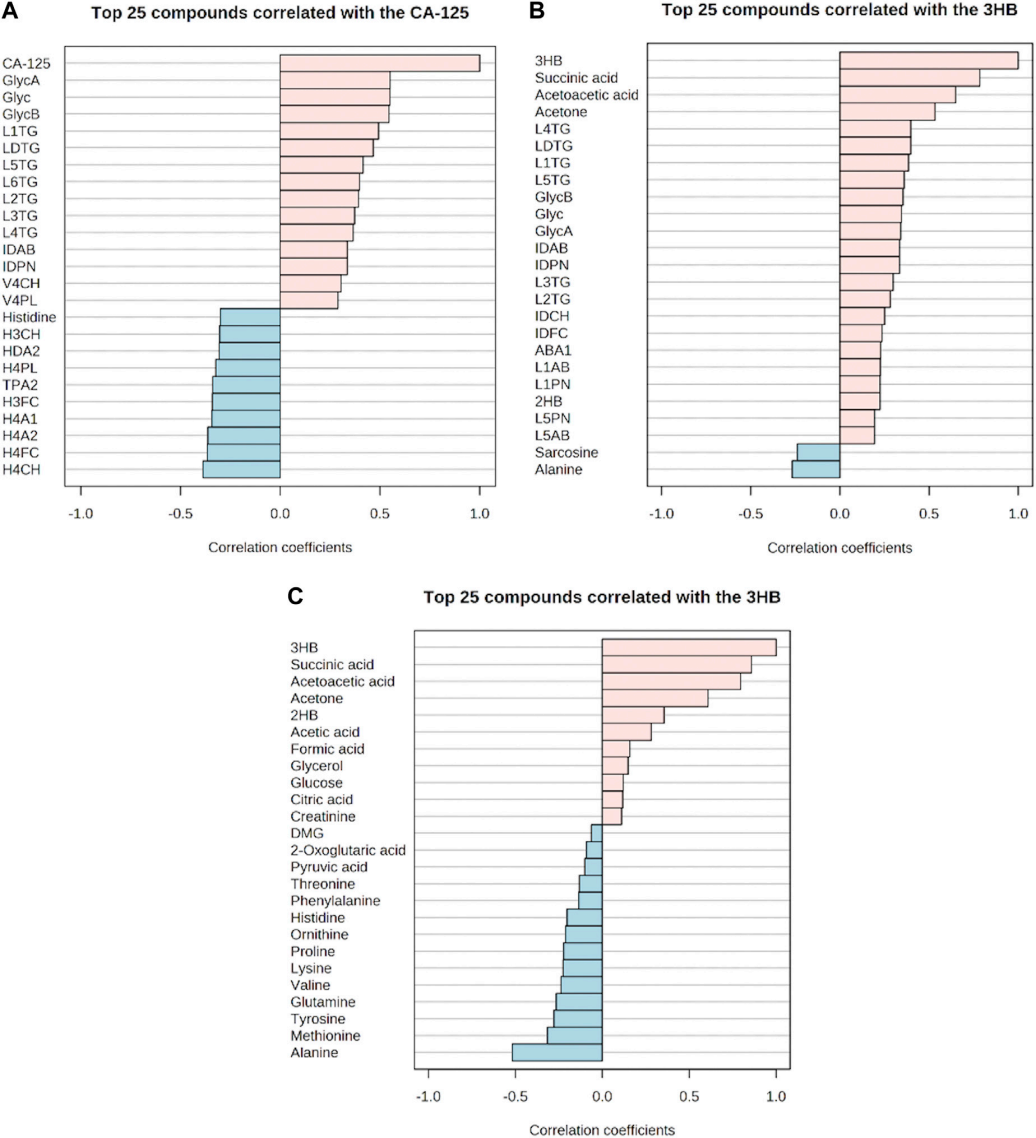
Correlation of the glycoprotein inflammation markers with metabolites, lipoproteins, and cancer antigen-125 markers. **(A–C)** Respective positive and negative correlations. **(A)** Positive and negative correlations of cancer antigen-125 with metabolite, lipoproteins, and inflammation markers. **(B)** Positive and negative correlations of 3-hydroxybutyric acid with metabolites, lipoproteins, and inflammation markers. **(C)** Positive and negative correlations of 3-hydroxybutyric acid with other metabolites.

Inflammation was positively correlated with ketone bodies (3-hydroxybutyric acid and acetoacetic acid), succinic acid, 2-hydroxybutyric acid, CA-125, and various parameters, mainly triglycerides, in lipoprotein fractions (LDLs) (Figures 7A, B). A negative correlation was observed for histidine, alanine, TPA2 (total plasma apolipoprotein A2), and subfraction of HDLs with certain lipid species, notably HDL-4 (Figure 7A). Moreover, two ketone bodies were positively correlated only with the inflammation markers and negatively correlated with alanine and sarcosine (Figures 7B, C). We also observed that the correlation between glycoprotein inflammation, CEA, and CA 19-9 antigen markers was weak (Supplementary Figures S8A, S8B).

## 4 Discussion

### 4.1 Alterations of metabolites and glycoprotein inflammation markers in borderline ovarian tumor and high-grade serous ovarian cancer clinical stages I–IV implicate critical roles in tumor development

It has been observed that malignant OC cells can disseminate to periglandular regions and the visceral omentum majus that is basically a large layer of adipocyte tissue (Lengyel et al., 2018). By the presence of a lesion in the omentum majus, these cells can make use of free fatty acids deriving from the adipocytes and switch from the glycolytic pathway into lipid metabolism where elevated fatty acid oxidation takes place for energy supply and tumor development (Balaban et al., 2017; Wu et al., 2019). It is furthermore consistent that we observed increased ketogenesis in HGSOC along with upregulated glycerol and glutamate. Elevated ketogenesis in the OC serum implicates the utilization of fatty acid (Braicu et al., 2017; Hilvo et al., 2016), reverse Warburg effect of circulating CAFs (Schauer et al., 2011; Ao et al., 2015), and cachexic phenotype (Pin et al., 2018), since the rate of hepatic fatty acid oxidation and fatty acid oxidation-related enzymes decreases along with hepatic ketogenesis and plasma ketone concentrations during acute phase response (Khovidhunkit et al., 2004b; Memon et al., 1992). Upregulated glycerol and glutamate represent an elevated rate of lipolysis in the adipocytes (Castelli et al., 2021; Nieman et al., 2011) and glutathione production (Aggarwal et al., 2019; Fazzari et al., 2015), respectively. As a consequence of a generally higher antioxidant capacity in the cancer cells, reactive oxygen species (ROS) do not induce apoptosis, but instead provoke inflammation, leading to facilitation in tumor development (Liou and Storz, 2010). In our results, the positive correlation of ketone bodies and highly observed NMR-based inflammatory markers of GlycA, GlycB, and Glyc in HGSOC further support this explanation.

2-Hydroxybutyric acid, a marker for insulin resistance (IR) and impaired glucose metabolism due to increased lipid oxidation and ROS (Gall et al., 2010), was higher in HGSOC. 2-Hydroxybutyrate was also higher in metastatic OC than in primary OC (Fong et al., 2011). Hence, the reason for increased glucose in HGSOC may not only be due to overexpression of GLUT1 (Lamkin et al., 2009), but it may be also attributed by IR that reduced the ability of skeletal, muscle, fat, and hepatic cells to take glucoses from the blood in response to normal circulating levels of insulin (Schwartsburd, 2019).

One of the hallmarks of cancer and key process in metastasis is the invasiveness of tumor cells (Hanahan and Weinberg, 2000). BOT has been characterized by the absence of stromal invasion and a less aggressive behavior compared to HGSOC (Brown et al., 2007); e.g., an increase in the circulating levels of formic acid or formate has been associated with an elevated rate of serine catabolism that takes place to promote invasiveness in oxidative glioblastoma multiforme cells (Meiser et al., 2018) and tumor progression in colorectal cancer (VanHook, 2022). Hence, the OC invasiveness may be facilitated by formate, which could explain why formate was higher over the clinical stages in this project.

Next, when cancer cells face genotoxic, oxidative, or nutritional stresses, they switch to amino acid metabolism guaranteeing their survival and proliferation (Wei et al., 2021). Decreased levels of alanine in HGSOC could be due to increased systemic inflammation as sustained systemic inflammation leads to hepatic glucose production followed by hyperglycemia in which the liver consumes alanine to perform gluconeogenesis and release acute phase response proteins (Gabay and Kushner, 1999; Okin and Medzhitov, 2016). Such phenomena could be linked to higher concentrations of 2-hydroxybutyric acid and glucose in HGSOC, increased phenylalanine levels in HGSOC by the systemic inflammation-induced influence of phenylalanine hydroxylase (Neurauter et al., 2008), the negative correlation between inflammation and alanine along with elevated ketogenesis, and decreased alanine levels which at the same time increased inflammation over the clinical stages. Moreover, the decreased level of alanine could reflect high glutamine uptake *via* alanine–serine–cysteine transporter 2 (ASCT2) (Guo et al., 2018).

The upregulation of excitatory amino acid (EAA) transporters is one of the characteristics of many cancers (Karunakaran et al., 2008; Saito and Soga, 2021). Decreased levels of histidine in HGOSC were reflective of the upregulation of EAA transporters, to meet requirements for their tumor development. Histidine was not only shown to be involved in cancer progression but also to be a metabolite which possesses anti-inflammatory properties (Grohmann and Bronte, 2010). One study showed a chemokine IL-8 response in a TNF-α-stimulated human leukemia monocytic cell line (THP-1) which was inhibited by histidine (Hasegawa et al., 2012). Furthermore, the production of TNF-α and IL-6 of lipopolysaccharide-induced mouse peritoneal macrophages was affected by histidine (Andou et al., 2009). Hence, the increase in inflammation parameters could be facilitated by a low level of histidine, as observed in the clinical stage-based comparison, with a negative correlation of histidine, CA-125, and glycoprotein inflammation markers.

### 4.2 Altered lipoproteins and glycoprotein inflammation markers in borderline ovarian tumor and high-grade serous ovarian cancer with clinical stages I–IV can be used to characterize tumor development and correlate to each other

Several researchers have reported about the altered lipoprotein profile of OC and ovarian tumor (OT) compared to healthy subjects. For example, TC levels were lower in OT (Melvin et al., 2012), and also, HDLs decreased in OT (Camps et al., 2021; Gadomska et al., 2005). Furthermore TG, HDCH, CL, and LDCH decreased (Qadir and Malik, 2008) in OC patients. In this project, lipoprotein profiles were investigated based on histology and clinical stages in order to observe which lipoproteins could contribute to the development of OC.

Increased levels of VLDL in OC patients were observed by Manisha and Jindal (2019) and Tiwari et al. (2015), which is consistent with the results in this project where we identified VLPN, VLAB, V4CH, V4PL, and V4TG to be higher in HGSOC. Such increased lipoproteins indicate that CL, phospholipids, and TG were transferred to HGSOC cells. Moreover, it has been found that LDL receptors (LDLRs) are overexpressed by many tumors (Rensen et al., 2001) and upregulated in OC patients in relation to healthy subjects (Jaragh Alhadad, 2021), implicating that non-significant TG and CL may be due to elevated consumption of the tumor development.

In the clinical stages, most of the altered lipoproteins were observed in the same manner as in a histology-based comparison where V5TG and ABA1 also increased over the clinical stages. Moreover, altered HDLs were clearly shown as depicted by the levels of H3FC, H4A1, H4A2, H4FC, HDA1, HDCH, TPA1 (total plasma apolipoprotein A1), and TPA2 decreased. It has been discovered that inflammation is characterized by increased LDLs and TG, ApoB, and decreased HDLs in chronic diseases (Tsoupras et al., 2018). The reason why they decreased may be due to inflammation-associated mechanisms. First, serum amyloid A (SAA) production increases in the liver by which SAA bind to HDLs to displace apoA-1 and apoA-2 for HDL clearance (Benditt and Eriksen, 1977; Eriksen and Benditt, 1980; Hosoai et al., 1999; Ashby et al., 2001). Second, SAA decreases the level of apoA-1 and apoA-2 HDLs (Benditt and Eriksen, 1977; Eriksen and Benditt, 1980), affecting the synthesis of HDLs (Florea et al., 2022). Third, the synthesis of apoA-1 decreases in the liver, leading to a decrease in HDL levels (Khovidhunkit et al., 2004a). Last, inflammation induces VLDL production and secretion in the liver, and decreases the hepatic clearance of TG-rich lipoproteins (Feingold et al., 2021). Indeed, such mechanisms and findings can describe the negative correlation between the Glyc inflammation markers, HDLs and TPA2. Moreover, this is in accordance with our findings that subfractions of VLDLs, IDLs, and LDLs with certain lipid species and apolipoprotein B-100 were increased in advanced OC and were positively correlated with Glyc inflammation and CA-125 markers.

Following decreased HDLs, cancer cells can maintain CL homeostasis, carry out angiogenesis, and escape immune surveillance (Ossoli et al., 2022). Additionally, oxidation of LDLs takes place more often, promoting the production of TG along with an accumulation of fatty acids in the adipocytes (Merkel et al., 2002). In other words, the transportation of fatty acids from the adipocytes to OC cells may be also facilitated by these discovered altered lipoproteins.

Another finding of this project was that ketogenesis was positively correlated with succinic acid that can be seen as increased marker during inflammation. Such correlations may indicate that OC cells utilized glutamine and fatty acids to produce glutathione and acquire ATP through the tricarboxylic acid (TCA) cycle, respectively, while potentially sparing glucose. The increase in succinate during inflammation could be due to the fact that this metabolite is a pro-inflammatory agent inducing IL-1β through HIF-1α in macrophages (Tannahill et al., 2013). In turn, metastasis of OC cells could be facilitated by the IL-1β/β1 integrin axis (Watanabe et al., 2012), and inflammation-associated cells transformed into cancer-associated immune cells (Bent et al., 2018) that further developed OC proliferation, invasion, and metastasis.

### 4.3 The NMR-based inflammation markers GlycA, GlycB, and Glyc are potential candidates for future diagnosis, prognosis, and treatment response of ovarian cancer

We observed that NMR parameters themselves could not improve diagnostic sensitivity and specificity compared to CA-125 alone. Yet, we found that CA-125/sarcosine, CA-125/pyruvate, CA-125/3HB, and CA-125/oxoglutaric acid could be potential biomarkers. These metabolites are involved in OC proliferation (Yuan et al., 2015), invasiveness with resistance to anoikis (Caneba et al., 2012), and one-carbon metabolism (Rizzo et al., 2018). Additionally, CA-125/3HB is indeed promising, since the elevated level of 3HB is reflective of the cachexic phenotype (Pin et al., 2018) and circulating CAFs in the blood (Schauer et al., 2011; Ao et al., 2015).

We showed that NMR-based inflammation parameters increased in advanced OC serum, indicating the elevated glycosylation of the acute phase proteins, such as α1-acid glycoprotein, haptoglobin, α1-antitrypsin, α1-antichymotrypsin, and transferrin (Otvos et al., 2015). Several studies confirm that haptoglobin β-chain (Ahmed et al., 2004; Ahmed et al., 2005), α1-acid glycoprotein (Rodríguez, 2019), α1-antitrypsin (Normandin et al., 2010), and α1-antichymotrypsin (Saldova et al., 2007) increased, and transferrin, the negative acute phase protein, decreased in OC during inflammation (Watanabe et al., 2014). Hence, haptoglobin β-chain, α1-acid glycoprotein, α1-antitrypsin, and α1-antichymotrypsin could be the inflammatory proteins that may contribute to the NMR peaks of GlycA, GlycB, and Glyc in OT and OC. Furthermore, such markers may be able to classify OC patients with and without ascites, since the presence of ascites arises by increased permeability of small vessels along with peritoneal parietal revascularization and glycoprotein production (Yung and Chan, 2011).

As observed in the results, Glyc inflammation and CA-125 markers behaved in the same way, which implicates that the effectiveness of radiotherapy and/or chemotherapy was low. Yet, CA-125 levels are influenced by a number of OC-unrelated conditions (Kobayashi et al., 2012). The cancer antigen marker is neither able to detect the early onset of OC (Journal, 2015) nor efficient in identifying asymptomatic OC patients (Skates et al., 2021), and 20% of OC have either low or completely missing presence of CA-125 (Journal, 2015). It is also observed that different kits and versions of the CA-125 test influence the absolute levels of CA-125 and test sensitivity (Kenemans et al., 1993), and the test sensitivity of OC deceases by more than 50% in the cutoff of the CA-125 level over 1,000 kU/l. (Moss et al., 2005). Of note, OC is not induced by CA-125, but inflammation. Several studies show that dysregulated inflammation is highly linked to tumor occurrence *via* angiogenesis and metastasis (Frantz et al., 2013; Qu et al., 2018; Zhao et al., 2018) and cancer-associated immune cells (Zhang et al., 2017). Moreover, the response of cancer to therapies is regulated by inflammation (Zhao et al., 2021). The response is either anti-tumor immunity *via* acute inflammation or therapy-elicited chronic inflammation along with subsequent therapeutic resistance and aggressive cancer progression (Zhao et al., 2021). In other words, Glyc inflammation markers are more reliable for cancer treatment outcomes. We could also stratify OC patients based on their quantitative inflammatory parameters, which clearly displayed specific alteration in metabolites, lipoproteins, and CA-125. Therefore, we conclude that CA-125/GlycA, CA-125/GlycB, and CA-125/Glyc, the use of both markers individually, and Glyc classes are potential for future diagnosis, prognosis, and treatment response of OC.

## 5 Conclusion

Profiles of metabolites, lipoproteins, and inflammation parameters of BOT and HGSOC serums were investigated using highly reproducible and quantitative IVDr NMR analysis. Hereby, we identified certain metabolites and lipoproteins that could be related to OC development along with acute and chronic inflammation. The NMR-based inflammation markers, GlycA, GlycB, and Glyc, were shown to be able to classify histology and early and advanced stages of ovarian cancer. Moreover, the ratios CA-125/GlycA, CA-125/GlycB, and CA-125/Glyc, the use of both markers individually, and Glyc classes could be an alternative to CA-125 alone for diagnosis, prognosis, and treatment response of EOC.

## 6 Declarations

• Ethics approval and consent to participate: 208/2021BO2
• Consent for publication: all authors read the manuscript and agree with its publication.
• Availability of data and materials: raw data are available upon request.
• Competing interests: GB, GeB, CT, NB, and SB report no competing interests.
• Funding: CT and GeB report research grants from Bruker BioSpin GmbH, Ettlingen, Germany.
• Authors’ contributions: conception: CT; design of the work: GB, GeB, and CT; data acquisition: GB, GeB, CC, HS, and SB; data analysis: GB and CT; data interpretation: GB, NB, and CT; figure preparation: GB; manuscript draft: GB, GeB, and CT; and manuscript editing, GB, GeB, AK, CC, HS, SK, SB, NB, and CT. All authors approved the submitted version.
• Acknowledgments: the authors want to thank the Werner Siemens Imaging Foundation and Bernd Pichler for supporting this project. The authors also thank Daniele Bucci for excellent technical assistance.

## Data Availability

The raw data supporting the conclusion of this article will be made available by the authors, without undue reservation.

## Glossary

Name: Extended Name
2HB: 2-hydroxybutyric acid
3HB: 3-hydroxybutyric acid
ABA1: apolipoprotein-B100/apolipoprotein A1
ApoE: apolipoprotein E
ASCT2: alanine–serine–cysteine transporter 2
ATP: adenosine triphosphate
BOT: borderline ovarian tumor
CA 19-9: cancer antigen 19-9
CA-125: cancer antigen-125
CAFs: cancer-associated fibroblasts
CEA: carcinoembryonic antigen
CL: cholesterols
DMG: N, N-dimethylglycine
EOC: epithelial ovarian cancer
FC: fold change
FDR: false discovery rate
GlcNAc: N-acetylgalactosamine
GLUT1: glucose transporter 1
GlycA: glycoprotein A or glycoprotein acetylation
H1A1: apolipoprotein-A1 HDL-1
H1A2: apolipoprotein-A2 HDL-1
H1CH: cholesterol HDL-1
H1FC: free cholesterol HDL-1
H1PL: phospholipids HDL-1
H1TG: triglycerides HDL-1
H2A1: apolipoprotein-A1 HDL-2
H2A2: apolipoprotein-A2 HDL-2
H2CH: cholesterol HDL-2
H2FC: free cholesterol HDL-2
H2PL: phospholipids HDL-2
H2TG: triglycerides HDL-2
H3A1: apolipoprotein-A1 HDL-3
H3A2: apolipoprotein-A2 HDL-3
H3CH: cholesterol HDL-3
H3FC: free cholesterol HDL-3
H3PL: phospholipids HDL-3
H3TG: triglycerides HDL-3
H4A1: apolipoprotein-A1 HDL-4
H4A2: apolipoprotein-A2 HDL-4
H4CH: cholesterol HDL-4
H4FC: free cholesterol HDL-4
H4PL: phospholipids HDL-4
H4TG: triglycerides HDL-4
HDA1: HDL-apolipoprotein A1
HDA2: HDL-apolipoprotein-A2
HDCH: HDL cholesterol
HDFC: HDL-free cholesterol
HDPL: HDL phospholipids
HDTG: HDL triglycerides
HGSOC: high-grade serous ovarian cancer or carcinoma
HIF: hypoxia-inducible factor
HK2: hexokinase 2
IDAB: IDL-apolipoprotein-B100
IDCH: IDL cholesterol
IDFC: IDL-free cholesterol
IDPL: IDL phospholipids
IDPN: ILDL particle number
IDTG: IDL triglycerides
IR: insulin resistance
KNN: K-nearest neighbors
L1AB: apolipoprotein-B100 LDL-1
L1CH: cholesterol LDL-1
L1FC: free cholesterol LDL-1
L1PL: phospholipids LDL-1
L1PN: particle number LDL-1
L1TG: triglycerides LDL-1
L2AB: apolipoprotein-B100 LDL-2
L2CH: cholesterol LDL-2
L2FC: free cholesterol LDL-2
L2PL: phospholipids LDL-2
L2PN: particle number LDL-2
L2TG: triglycerides LDL-2
L3AB: apolipoprotein-B100 LDL-3
L3CH: cholesterol LDL-3
L3FC: free cholesterol LDL-3
L3PL: phospholipids LDL-3
L3PN: particle number LDL-3
L3TG: triglycerides LDL-3
L4AB: apolipoprotein-B100 LDL-4
L4CH: cholesterol LDL-4
L4FC: free cholesterol LDL-4
L4PL: phospholipids LDL-4
L4PN: particle number LDL-4
L4TG: triglycerides LDL-4
L5AB: apolipoprotein-B100 LDL-5
L5CH: cholesterol LDL-5
L5FC: free cholesterol LDL-5
L5PL: phospholipids LDL-5
L5PN: particle number LDL-5
L5TG: triglycerides LDL-5
L6AB: apolipoprotein-B100 LDL-6
L6CH: cholesterol LDL-6
L6FC: free cholesterol LDL-6
L6PL: phospholipids LDL-6
L6PN: particle number LDL-6
L6TG: triglycerides LDL-6
LDAB: LDL-apolipoprotein-B100
LDCH: LDL cholesterol
LDFC: LDL-free cholesterol
LDH: lactate dehydrogenase
LDHD: LDL-cholesterol/HDL-cholesterol
LDLR: LDL receptor
LDPL: LDL phospholipids
LDPN: LDL particle number
LDTG: LDL triglycerides
LOOCV: leave one out cross-validation method
LPL: lipoprotein lipase
MCTs: lactate transporter (monocarboxilate transporter)
NANA: N-acetylneuraminic acid
OC: ovarian cancer
PCA: principle component analysis
PCOS: polycystic ovary syndrome
PKM2: pyruvate kinase type M2
ROC: receiver operating characteristic
ROS: reactive oxygen species
SAA: serum amyloid A
sPLSDA: sparse partial least square discriminant analysis
TBPN: total particle number (apolipoprotein-B100 carrying particles)
TCA: tricarboxylic acid cycle
TG: triglycerides
THP-1: human leukemia monocytic cell line
TME: tumor microenvironment
TPA1: total plasma apolipoprotein A1
TPA2: total plasma apolipoprotein-A2
TPAB: total plasma apolipoprotein-B100
TPCH: total plasma cholesterol
TPTG: total plasma triglyceride
V1CH: cholesterol VLDL-1
V1FC: free cholesterol VLDL-1
V1PL: phospholipids VLDL-1
V1TG: triglycerides VLDL-1
V2CH: cholesterol VLDL-2
V2FC: free cholesterol VLDL-2
V2PL: phospholipids VLDL-2
V2TG: triglycerides VLDL-2
V3CH: cholesterol VLDL-3
V3FC: free cholesterol VLDL-3
V3PL: phospholipids VLDL-3
V3TG: triglycerides VLDL-3
V4CH: cholesterol VLDL-4
V4FC: free cholesterol VLDL-4
V4PL: phospholipids VLDL-4
V4TG: triglycerides VLDL-4
V5CH: cholesterol VLDL-5
V5FC: free cholesterol VLDL-5
V5PL: phospholipids VLDL-5
V5TG: triglycerides VLDL-5
VLAB: VLDL-apolipoprotein-B100
VLCH: VLDL cholesterol
VLFC: VLDL-free cholesterol
VLPL: VLDL phospholipids
VLPN: VLDL particle number
