# Supplementary material for: Stratification of ovarian cancer borderline from high-grade serous carcinoma patients by quantitative serum NMR spectroscopy of metabolites, lipoproteins, and inflammatory markers
Source: Front Mol Biosci. 2023 Apr 19;10:1158330. doi: 10.3389/fmolb.2023.1158330 (PMC10166069; doi:10.3389/fmolb.2023.1158330)
Supplement: Supplementary file 1 [file DataSheet1.pdf]

Supplementary Material

Stratification of ovarian cancer borderline from high-grade serous carcinoma patients by quantitative serum NMR spectroscopy of metabolites, lipoproteins and inflammatory markers

Gyuntae Bae[1], Georgy Berezhnoy[1], André Koch[2], Claire Cannet[3], Hartmut Schäfer[3], Stefan Kommoss[2], Sara Brucker[2], Nicolas Beziere[1,4], Christoph Trautwein[1,*]

[1] Werner Siemens Imaging Center, Department of Preclinical Imaging and Radiopharmacy, University Hospital Tübingen, Tübingen, Germany

[2] Department of Women's Health, University Hospital Tübingen, Tübingen, Germany

[3] Bruker BioSpin GmbH, Ettlingen, Germany

[4] Cluster of Excellence CMFI (EXC 2124) "Controlling Microbes to Fight Infections", Eberhard Karls University of Tübingen, Tübingen, Germany

*** Correspondence:** Christoph.Trautwein@med.uni-tuebingen.de

Supplementary Materials

Supplementary Table 1 – p-value and FDR of the significant metabolites in borderline ovarian tumor and high-grade serous ovarian cancer by volcano plot.

	FC	log2(FC)	raw.pval	-log10(p)	FDR
**Acetone (mmol/L)**	0.6675	-0.5832	0.0004	3.3961	0.0061
**3-Hydroxybutyric acid (mmol/L)**	0.5722	-0.8054	0.0006	3.2407	0.0061
**Acetoacetic acid (mmol/L)**	0.33713	-1.5686	0.0010	2.9836	0.0083
**2-Hydroxybutyric acid (mmol/L)**	0.3621	-1.4656	0.0048	2.3199	0.0219
**Glutamate (mmol/L)**	0.7870	-0.3457	0.0352	1.4536	0.1251
**Glycerol (mmol/L)**	0.7009	-0.5128	0.0473	1.3254	0.1513

Supplementary Table 2 – p-value and FDR of the significant metabolites in borderline ovarian tumor and high-grade serous ovarian cancer by comparative statistics

	raw.pval	FDR
**Histidine (mmol/L)**	0.0014	0.0087
**Glucose (mmol/L)**	0.0013	0.0087
**Alanine (mmol/L)**	9.4376E-5	0.0031
**Phenylalanine (mmol/L)**	0.0080	0.0644

Supplementary Table 3 – Mean and standard deviation of the significant metabolites in comparing histology of ovarian cancer.

	HGSOC (N = 123)	BOT (N = 47)
**Acetone (mmol/L)**	0.0482±0.0483	0.03217±0.0337
**3-Hydroxybutyric acid (mmol/L)**	0.5722±0.3546	0.1528±0.2263
**Acetoacetic acid (mmol/L)**	0.07920±0.1346	0.02670±0.0449
**2-Hydroxybutyric acid (mmol/L)**	0.03862±0.0676	0.01398±0.0439
**Glutamate (mmol/L)**	0.0691±0.0472	0.05438±0.0361
**Glycerol (mmol/L)**	0.2028±0.1803	0.1421±0.1532
**Histidine (mmol/L)**	0.0741±0.0176	0.0841±0.0194
**Glucose (mmol/L)**	6.289±1.469	5.579±1.117
**Alanine (mmol/L)**	0.3884±0.0917	0.4513±0.0915
**Phenylalanine (mmol/L)**	0.05335±0.0470	0.01448±0.01219

Supplementary Table 4 – p-value and FDR of the significant lipoproteins in borderline ovarian tumor and high-grade serous ovarian cancer by volcano plot.

	FC	log2(FC)	raw.pval	-log10(p)	FDR
**L1TG (mg/dL)**	0.7058	-0.5027	3.5063E-07	6.4551	2.4878E-05
**LDTG (mg/dL)**	0.7557	-0.4041	4.4426E-07	6.3524	2.4878E-05

**L2TG (mg/dL)**	0.7544	-0.4065	1.9868E-06	5.7018	7.4173E-05
**L4TG (mg/dL)**	0.7226	-0.4688	3.0732E-05	4.5124	0.0007
**L5TG (mg/dL)**	0.7572	-0.4012	3.2403E-05	4.4894	0.0007
**IDAB (mg/dL)**	0.7592	-0.3975	9.6321E-05	4.0163	0.0015
**IDPN (nmol/L)**	0.7592	-0.3974	9.6340E-05	4.0162	0.0015
**H2TG (mg/dL)**	0.7873	-0.3450	0.0003	3.5735	0.0037
**L3TG (mg/dL)**	0.8256	-0.2765	0.0003	3.5122	0.0038
**L6TG (mg/dL)**	0.8144	-0.2962	0.0004	3.3915	0.0040
**V4PL (mg/dL)**	0.7358	-0.4425	0.0004	3.366	0.0040
**V4CH (mg/dL)**	0.7223	-0.4694	0.0005	3.2927	0.0043
**H1TG (mg/dL)**	0.7951	-0.3308	0.0006	3.2564	0.0043
**V4TG (mg/dL)**	0.7343	-0.4456	0.0006	3.2396	0.0043
**V4FC (mg/dL)**	0.7134	-0.4872	0.0015	2.8184	0.0100
**VLAB (mg/dL)**	0.7638	-0.3888	0.0017	2.7643	0.0102
**VLPN (nmol/L)**	0.7638	-0.3887	0.0017	2.7617	0.0102
**IDCH (mg/dL)**	0.8084	-0.3069	0.0049	2.6327	0.0215
**IDFC (mg/dL)**	0.8004	-0.3213	0.0050	2.5904	0.0215
**V3PL (mg/dL)**	0.7810	-0.3566	0.0213	1.671	0.0771
**V5CH (mg/dL)**	0.7865	-0.3466	0.0245	1.6434	0.0831
**V3CH (mg/dL)**	0.7690	-0.3790	0.0259	1.5866	0.0842
**V3FC (mg/dL)**	0.7598	-0.3964	0.0268	1.5712	0.0842
**V5FC (mg/dL)**	0.8206	-0.2852	0.0360	1.3253	0.1185

Supplementary Table 5 – Mean and standard deviation of the significant lipoproteins in comparing histology of ovarian cancer.

	**HGSOC (N = 122)**	**BOT (N = 48)**
**L1TG (mg/dL)**	8.792±3.589	6.205±2.472
**LDTG (mg/dL)**	28.58±9.370	21.59±6.869
**L2TG (mg/dL)**	3.512±1.251	2.649±0.9380
**L4TG (mg/dL)**	3.576±1.596	2.584±1.155
**L5TG (mg/dL)**	3.763±1.410	2.849±1.394
**IDAB (mg/dL)**	7.447±2.569	5.654±2.589
**IDPN (mg/dL)**	135.4±46.70	102.8±47.08
**H2TG (mg/dL)**	2.137±0.6574	1.682±0.7590
**L3TG (mg/dL)**	3.494±1.040	2.885±0.9029
**L6TG (mg/dL)**	5.972±1.948	4.863±1.839
**V4PL (mg/dL)**	5.581±2.501	4.106±2.061
**V4CH (mg/dL)**	6.380±3.046	4.608±2.539
**H1TG (mg/dL)**	4.942±1.705	3.930±2.265
**V4TG (mg/dL)**	11.21±5.268	8.231±4.036
**V4FC (mg/dL)**	2.782±1.524	1.985±1.202
**VLAB (mg/dL)**	9.500±4.311	7.256±3.455
**VLPN (mg/dL)**	172.7±78.38	131.9±62.83
**IDCH (mg/dL)**	17.49±6.602	14.14±7.634

**IDFC (mg/dL)**	4.875±1.947	3.901±2.154
**V3PL (mg/dL)**	4.419±2.447	3.451±1.832
**V5CH (mg/dL)**	1.286±0.7346	1.012±0.6443
**V3CH (mg/dL)**	4.396±2.579	3.381±1.895
**V3FC (mg/dL)**	1.893±1.181	1.439±0.8821
**V5FC (mg/dL)**	0.9080±0.4623	0.7451±0.4250

Supplementary Table 6 – p-value and FDR of glycoprotein inflammation and CA-125 markers in comparing histology of ovarian cancer.

	**raw.pval**	**FDR**
**GlycA (p.d.u)**	1.0736E-08	1.2791E-08
**Glyc (p.d.u)**	1.2726E-08	1.2791E-08
**GlycB (p.d.u)**	1.2791E-08	1.2791E-08
**CA-125 (kU/l)**	4.4748E-11	1.7899E-10

Supplementary Table 7 – Mean and standard deviation of glycoprotein inflammation and CA-125 markers in comparing histology of ovarian cancer.

	**HGSOC (N = 121)**	**BOT (N = 46)**
**GlycA (p.d.u)**	1.198±0.2319	0.9761±0.1797
**GlycB (p.d.u)**	0.4557±0.0866	0.3735±0.0666
**Glyc (p.d.u)**	1.653±0.3184	1.350±0.2458
**CA-125 (kU/l)**	945.5±1675	160.2±571.4

Supplementary Table 8 – p-value and FDR of the significant metabolites in ovarian tumor and cancer with clinical stages I-IV by comparative statistics.

	**raw.pval**	**FDR**
**Histidine (mmol/L)**	9.4324E-5	0.0031
**Acetoacetic acid (mmol/L)**	0.0013	0.0213
**Alanine (mmol/L)**	0.0010	0.0116
**Formic acid (mmol/L)**	0.0028	0.0227

Supplementary Table 9 – Mean and standard deviation of the significant metabolites in comparing ovarian tumor and cancer with clinical stages I-IV.

	**I (N = 43)**	**II (N = 12)**	**III (N = 67)**	**IV (N = 27)**
**Histidine (mmol/L)**	0.0815±0.0203	0.0807±0.0138	0.0785±0.0177	0.0622±0.0138
**Acetoacetic acid (mmol/L)**	0.03238±0.0502	0.1757±0.2620	0.05802±0.0849	0.1077±0.1648
**Alanine (mmol/L)**	0.4297±0.0974	0.3866±0.1001	0.4144±0.0889	0.3421±0.0813
**Formic acid (mmol/L)**	0.0194±0.0066	0.0195±0.0109	0.0227±0.0077	0.0248±0.0068

Supplementary Table 10 – p-Value and FDR of the significant lipoproteins in ovarian tumor and cancer with clinical stages I-IV by comparative statistics.

	p.value	FDR
**L1TG (mg/dL)**	8.3197E-07	9.3181E-05
**LDTG (mg/dL)**	3.7018E-06	0.0002
**L2TG (mg/dL)**	4.8378E-05	0.0015
**L5TG (mg/dL)**	6.6134E-05	0.0015
**L4TG (mg/dL)**	0.0011	0.0127
**V4TG (mg/dL)**	4.4006E-4	0.0127
**V4PL (mg/dL)**	4.8788E-4	0.0127
**V5TG (mg/dL)**	0.0011	0.0127
**L3TG (mg/dL)**	0.0057	0.0280
**H4FC (mg/dL)**	0.0039	0.0275
**H4CH (mg/dL)**	0.0014	0.0135
**V4CH (mg/dL)**	0.0014	0.0135
**IDAB (mg/dL)**	0.0011	0.0086
**IDPN (nmol/L)**	0.0011	0.0086
**VLPN (nmol/L)**	9.028E-4	0.0127
**VLAB (mg/dL)**	9.006E-4	0.0127
**H1TG (mg/dL)**	0.0051	0.0280
**H2TG (mg/dL)**	0.0011	0.0127
**H3FC (mg/dL)**	0.0047	0.0280
**HDTG (mg/dL)**	0.0042	0.0275
**V5FC (mg/dL)**	0.0050	0.0280
**H4A2 (mg/dL)**	0.0055	0.0280
**V4FC (mg/dL)**	0.0057	0.0280
**V5CH (mg/dL)**	0.0061	0.0282
**TPA2 (mg/dL)**	0.0061	0.0273
**H4A1 (mg/dL)**	0.0104	0.0465
**V5PL (mg/dL)**	0.0128	0.0552
**L6TG (mg/dL)**	0.0137	0.0567
**HDA1 (mg/dL)**	0.0209	0.0709
**TPA1 (mg/dL)**	0.0181	0.0650
**IDCH (mg/dL)**	0.0186	0.0650
**HDCH (mg/dL)**	0.013008	0.0443
**V3PL (mg/dL)**	0.0166	0.0619
**IDFC (mg/dL)**	0.0224	0.0718
**ABA1 (mg/dL)**	0.01559	0.0499

Supplementary Table 11 – Mean and standard deviation of significant lipoproteins in comparing ovarian tumor and cancer with clinical stages I-IV.

	I (N = 42)	II (N = 12)	III (N = 68)	IV (N = 26)
**L1TG (mg/dL)**	6.585±3.605	8.265±4.799	8.012±2.438	10.48±3.180

**LDTG (mg/dL)**	22.90±10.01	27.01±11.48	7.04426.99±	32.99±8.621
**L2TG (mg/dL)**	2.833±1.331	3.274±1.196	3.284±1.065	3.982±1.030
**L5TG (mg/dL)**	2.979±1.582	3.477±1.511	3.637±1.325	4.469±1.279
**L4TG (mg/dL)**	2.807±1.730	3.240±1.523	3.362±1.330	4.318±1.388
**V4TG (mg/dL)**	7.756±4.113	10.02±6.096	11.24±4.630	12.46±5.945
**V4PL (mg/dL)**	3.867±2.060	5.103±2.989	5.629±2.287	6.068±2.770
**V5TG (mg/dL)**	2.575±0.7980	3.191±1.476	3.180±0.9631	3.520±1.064
**L3TG (mg/dL)**	2.982±1.216	3.430±1.086	3.340±0.8438	3.879±0.9524
**H4FC (mg/dL)**	4.829±1.311	4.453±1.383	4.293±1.370	3.634±0.9709
**H4CH (mg/dL)**	22.08±5.151	21.16±5.016	19.46±4.745	17.52±4.142
**V4CH (mg/dL)**	4.405±2.562	5.637±3.284	6.522±2.917	6.754±3.340
**IDAB (mg/dL)**	5.739±2.900	7.184±3.363	7.379±2.213	7.962±2.647
**IDPN (nmol/L)**	104.4±52.73	130.6±61.15	134.2±40.24	144.8±48.13
**VLPN (nmol/L)**	124.5±61.91	155.3±99.10	170.0±68.12	195.8±88.79
**VLAB (mg/dL)**	6.845±3.405	8.543±5.449	9.352±3.746	10.77±4.883
**H1TG (mg/dL)**	3.949±2.188	4.108±1.591	5.183±1.798	4.440±1.268
**H2TG (mg/dL)**	1.655±0.7198	1.853±0.7438	2.182±0.6559	2.081±0.5938
**H3FC (mg/dL)**	3.203±0.8712	3.001±1.016	3.006±0.9385	2.405±0.6840
**HDTG (mg/dL)**	10.03±3.550	10.48±3.707	12.35±3.216	11.42±2.892
**V5FC (mg/dL)**	0.6666±0.3940	1.035±0.3535	0.9681±0.5116	0.8254±0.4218
**H4A2 (mg/dL)**	20.78±5.304	19.68±4.296	18.56±4.963	16.54±3.810
**V4FC (mg/dL)**	1.888±1.229	2.571±1.774	2.874±1.441	2.869±1.722
**V5CH (mg/dL)**	0.8923±0.5791	1.445±0.6832	1.305±0.7219	1.397±0.8414
**TPA2 (mg/dL)**	38.28±7.460	37.58±7.705	37.20±7.637	32.54±5.691
**H4A1 (mg/dL)**	81.55±16.85	79.63±15.67	75.78±15.51	68.64±13.32
**V5PL (mg/dL)**	1.234±0.6233	1.752±0.6871	1.705±0.8456	1.752±0.9795
**L6TG (mg/dL)**	5.059±1.978	5.614±2.512	5.762±1.762	6.673±2.135
**HAD1 (mg/dL)**	180.6±37.40	179.6±38.00	181.1±36.07	156.6±28.14
**TPA1 (mg/dL)**	177.2±34.92	176.8±37.97	178.1±33.95	154.3±27.66
**IDCH (mg/dL)**	13.84±7.719	16.69±7.151	18.20±6.504	17.40±7.129
**HDCH (mg/dL)**	74.26±17.14	72.91±19.52	73.79±18.41	62.91±13.60
**V3PL (mg/dL)**	3.233±1.864	3.759±2.268	4.565±2.342	4.668±2.778
**IDFC (mg/dL)**	3.822±2.236	4.727±2.095	5.055±1.884	4.880±2.120
**ABA1**	0.5950±0.2395	0.6542±0.1939	0.6281±0.1801	0.7338±0.1920

Supplementary Table 12 – p-value and FDR of cancer antigen-125 on borderline ovarian tumor and high-grade serous ovarian cancer by comparative statistics.

	**raw.pval**	**FDR**

**CA-125 (kU/l)**	9.8824E-11	8.8941E-10

Supplementary Table 13 – Mean and standard deviation of cancer antigen-125 in comparing ovarian tumor and cancer with clinical stages I-IV.

	I (N = 41)	II (N = 12)	III (N = 70)	IV (N = 27)
**CA-125 (kU/l)**	99.93±235.4	300.3±421.3	1100±1755	1095±1899

Supplementary Table 14 – p-value and FDR of glycoprotein inflammation markers on borderline ovarian tumor and high-grade serous ovarian cancer by comparative statistics.

	raw.pval	FDR
**GlycA (p.d.u)**	4.3648E-08	7.3174E-08
**GlycB (p.d.u)**	5.128E-08	7.3174E-08
**Glyc (p.d.u)**	7.3174E-08	7.3174E-08

Supplementary Table 15 – Mean and standard deviation of glycoprotein inflammation markers in comparing ovarian tumor and cancer with clinical stage I-IV.

	I (N = 39)	II (N = 12)	III (N = 68)	IV (N = 26)
**GlycA (p.d.u)**	1.002±0.2143	1.122±0.2353	1.166±0.2067	1.346±0.2058
**GlycB (p.d.u)**	0.3849±0.0805	0.4250±0.0918	0.4432±0.0786	0.5119±0.0720
**Glyc (p.d.u)**	1.387±0.2930	1.546±0.3248	1.610±0.2852	1.857±0.2806

Supplementary Table 16 – Results of PLS-DA cross-validation.

Measure	1 comps	2 comps	3 comps
Accuracy	0.85619	0.85619	0.85619
R2	0.0073475	0.0131	0.014047
Q2	-0.034421	-0.060856	-0.077725

Supplementary Table 17 – Correlation of cancer antigen-125 with metabolites, lipoproteins and glycoprotein inflammation markers.

	correlation	t-stat	p-value	FDR
**CA-125**	1	0	0	0
**GlycA**	0.5511	306450	4.33E-14	2.76E-12
**Glyc**	0.5490	307840	5.60E-14	2.76E-12
**GlycB**	0.5459	309990	8.30E-14	3.07E-12
**L1TG**	0.4924	346490	3.75E-11	1.11E-09
**LDTG**	0.4659	364630	5.38E-10	1.33E-08
**L5TG**	0.4149	399410	4.90E-08	1.04E-06
**L6TG**	0.3975	411330	1.95E-07	3.61E-06
**L2TG**	0.3922	414940	2.92E-07	4.80E-06
**H4CH**	-0.3864	946400	4.50E-07	6.66E-06

**L3TG**	0.3735	427660	1.15E-06	1.54E-05
**L4TG**	0.3660	432760	1.94E-06	2.36E-05
**H4FC**	-0.3651	931840	2.07E-06	2.36E-05
**H4A2**	-0.3628	930290	2.42E-06	2.56E-05
**H4A1**	-0.3437	917230	8.62E-06	8.51E-05
**H3FC**	-0.3409	915370	1.03E-05	9.50E-05
**TPA2**	-0.3387	913880	1.18E-05	0.0001
**IDAB**	0.3370	452570	1.31E-05	0.0001
**IDPN**	0.3365	452900	1.35E-05	0.0001
**H4PL**	-0.3232	903290	3.06E-05	0.0002
**HDA2**	-0.3069	892160	7.89E-05	0.0006
**V4CH**	0.3046	474690	8.98E-05	0.0006
**H3CH**	-0.3032	889580	9.76E-05	0.0006
**Histidine**	-0.2992	886900	0.0001	0.0007
**V4PL**	0.2892	485230	0.0002	0.0012
**Alanine**	-0.2537	855840	0.0012	0.0057

Supplementary Table 18 – Correlation of 3-hydrobutyric acid with metabolites, lipoproteins and glycoprotein inflammation markers.

	**correlation**	**t-stat**	**p-value**	**FDR**
**3-Hydroxybutyric acid**	1	0	0	0
**Succinic acid**	0.7837	147650	1.69E-34	1.25E-32
**Acetoacetic acid**	0.6492	239510	1.64E-20	8.08E-19
**Acetone**	0.5325	319160	4.27E-13	1.58E-11
**L4TG**	0.3978	411090	1.90E-07	4.73E-06
**LDTG**	0.3977	411180	1.92E-07	4.73E-06
**L1TG**	0.3839	420570	5.40E-07	1.14E-05
**L5TG**	0.3604	436640	2.86E-06	5.29E-05
**GlycB**	0.3533	441430	4.58E-06	7.53E-05
**Glyc**	0.3446	447430	8.13E-06	0.0001
**GlycA**	0.3420	449200	9.60E-06	0.0001
**IDAB**	0.3355	453620	1.45E-05	0.0002
**IDPN**	0.3353	453740	1.46E-05	0.0002
**L3TG**	0.2984	478970	0.0001	0.0013
**L2TG**	0.2818	490300	0.0003	0.0030
**Alanine**	-0.2675	865220	0.0006	0.0058
**IDCH**	0.2508	511400	0.0014	0.0120
**Sarcosine**	-0.2385	845440	0.0024	0.0197
**IDFC**	0.2359	521600	0.0027	0.0208
**ABA1**	0.2289	526410	0.0036	0.0267
**L1AB**	0.2258	528510	0.0041	0.0280
**L1PN**	0.2253	528840	0.0042	0.0280
**2-Hydroxybutyric acid**	0.2243	529510	0.0043	0.0280
**L5PN**	0.1953	549350	0.0133	0.0765

**L5AB**	0.1953	549360	0.0134	0.0765

Supplementary Table 19 – Correlation of 3-hydrobutyric acid with the other metabolites.

	correlation	t-stat	p-value	FDR
**3-Hydroxybutyric acid**	1	0	0	0
**Succinic acid**	0.8575	116710	2.36E-50	3.89E-49
**Acetoacetic acid**	0.7955	167430	2.16E-38	2.37E-37
**Acetone**	0.6079	321070	1.49E-18	1.23E-17
**Alanine**	-0.5172	1242300	5.14E-13	3.39E-12
**2-Hydroxybutyric acid**	0.3552	527940	2.00E-06	1.10E-05
**Methionine**	-0.3150	1076700	2.87E-05	0.0001
**Acetic acid**	0.2817	588150	0.0002	0.0008
**Tyrosine**	-0.2783	1046700	0.0002	0.0009
**Glutamine**	-0.2643	1035200	0.0005	0.0016
**Valine**	-0.2366	1012500	0.0019	0.0057
**Lysine**	-0.2254	1003300	0.0031	0.0086
**Proline**	-0.2216	1000200	0.0037	0.0094
**Ornithine**	-0.2110	991570	0.0057	0.0135
**Histidine**	-0.2024	984560	0.0081	0.0178
**Formic acid**	0.1581	689320	0.0394	0.0813
**Glycerol**	0.1494	696440	0.0518	0.1005
**Phenylalanine**	-0.1352	929500	0.0788	0.1408
**Dimethylsulfone**	-0.1328	927510	0.0844	0.1408
**Threonine**	-0.1323	927170	0.0854	0.1408
**Glucose**	0.1213	719480	0.1151	0.1808
**Citric acid**	0.1178	722340	0.1260	0.1890
**Creatinine**	0.1103	728460	0.1520	0.2181
**Pyruvic acid**	-0.1007	901230	0.1915	0.2633
**2-Oxoglutaric acid**	-0.0907	893030	0.2397	0.3165

Supplementary Table 20 – Correlation of carcinoembryonic antigen with glycoprotein inflammation markers.

	correlation	t-stat	p-value	FDR
**CEA**	1	6.9051E-12	0	0
**GlycB**	0.2285	47981	0.0535	0.0734
**Glyc**	0.2202	48501	0.0631	0.0734
**GlycA**	0.2123	48993	0.0734	0.0734

Supplementary Table 21 – Correlation of cancer antigen 19-9 with glycoprotein inflammation markers.

	correlation	t-stat	p-value	FDR
**CA19-9**	1	0	0	0
**GlycA**	0.0387	133050	0.7109	0.7342

**Glyc**	0.0367	133340	0.7255	0.7342
**GlycB**	0.03549	133500	0.7342	0.7342

Supplementary Table 22 – Information of each Materials.

Material	Company	Catalog Number (LOT) / ID
5mm NMR tube	Bruker	4004151/1
Cap sealing ball	Bruker	Z147554
KIMTECH Tissue	KIMTECH^{science}	7558
Pipette &Pipette Tip	Eppendorf	S1111-6801
600 NMR	Bruker	Z127016
Eppendorf tube (1.5ml)	SARSTEDT AG & Co. KG	1082021
Bruker Plasma Buffer (100ml)	Bruker	H145686_01

Supplementary Table 23 – p-value and FDR of altered metabolites, lipoproteins and CA-125 markers in borderline ovarian tumor and high-grade serous ovarian cancer based on inflammatory concentration.

	p.value	FDR		p.value	FDR
**GlycA**	1.93E-69	1.54E-67	**H4A1**	6.04E-05	0.000234
**Glyc**	2.08E-69	1.54E-67	**H4PL**	6.17E-05	0.000234
**GlycB**	1.48E-67	7.28E-66	**TBPN**	7.74E-05	0.00028
**LDTG**	1.90E-15	7.01E-14	**TPAB**	7.74E-05	0.00028
**IDAB**	2.49E-13	6.21E-12	**Glutamate**	8.42E-05	0.000297
**IDPN**	2.52E-13	6.21E-12	**Alanine**	0.000272	0.000937
**CA-125**	6.73E-11	1.42E-09	**H3A1**	0.000289	0.000971
**L1TG**	1.18E-10	2.18E-09	**Acetone**	0.000337	0.001107
**L6TG**	1.77E-10	2.91E-09	**HDA1**	0.000346	0.001112
**L4TG**	2.42E-10	3.57E-09	**TPA1**	0.000634	0.001998
**L2TG**	1.01E-09	1.36E-08	**H3CH**	0.000724	0.002234
**L5TG**	3.36E-09	4.14E-08	**L6FC**	0.001066	0.00322
**V4CH**	5.95E-09	6.77E-08	**L5PL**	0.001337	0.003956
**ABA1**	6.77E-09	7.16E-08	**V3TG**	0.001665	0.004831
**IDCH**	8.95E-09	8.83E-08	**L1PN**	0.00175	0.004925
**V4PL**	1.94E-08	1.80E-07	**L1AB**	0.001764	0.004925
**V4TG**	2.30E-08	2.00E-07	**LDPN**	0.001921	0.00517
**IDFC**	4.26E-08	3.50E-07	**LDAB**	0.001921	0.00517
**VLAB**	1.29E-06	9.61E-06	**VLCH**	0.002115	0.00559
**VLPN**	1.30E-06	9.61E-06	**Acetoacetic acid**	0.002228	0.005786
**HDFC**	3.01E-06	2.12E-05	**LDHD**	0.002417	0.006138
**L6AB**	3.29E-06	2.12E-05	**Glycerol**	0.002447	0.006138

**L6PN**	3.30E-06	2.12E-05	**V2FC**	0.003212	0.007922
**Histidine**	5.58E-06	3.35E-05	**L5CH**	0.003558	0.008632
**L5AB**	5.86E-06	3.35E-05	**Phenylalanine**	0.00398	0.009501
**L5PN**	5.88E-06	3.35E-05	**H2TG**	0.004081	0.009588
**V3PL**	8.10E-06	4.44E-05	**H3FC**	2.44E-05	0.000109
**V4FC**	9.76E-06	5.16E-05	**H4CH**	2.64E-05	0.000115
**L6CH**	1.30E-05	6.63E-05	**V3CH**	4.18E-05	0.000177
**L6PL**	1.90E-05	9.36E-05	**Succinic acid**	4.73E-05	0.000192
**L3TG**	2.27E-05	0.000108	**H4FC**	4.79E-05	0.000192
**V3FC**	2.33E-05	0.000108			

Supplementary Table 24 – NMR parameters alone have only AUC 0.6-0.7 for classifying histology of ovarian cancer.

Name	AUC	T-tests	Log2 FC	Name	AUC	T-tests	Log2 FC
**L4TG**	0.719	0.000	-0.501	**Glycerol**	0.612	0.038	0.121
**IDPN**	0.698	0.000	-0.099	**ABA1**	0.609	0.050	0.280
**IDAB**	0.698	0.000	-0.263	**H3FC**	0.608	0.047	0.143
**H2TG**	0.695	0.000	-0.806	**L1PL**	0.605	0.061	-0.042
**Alanine**	0.690	0.000	-0.247	**L6PN**	0.603	0.033	-0.030
**H1TG**	0.686	0.000	-0.388	**L6AB**	0.603	0.033	-0.057
**H4CH**	0.682	0.000	0.075	**V3CH**	0.602	0.063	-0.271
**L6TG**	0.678	0.000	-0.201	**V5CH**	0.602	0.076	-99.000
**L3TG**	0.678	0.001	-0.258	**H3FC**	0.608	0.047	0.143
**H4A2**	0.673	0.001	0.074	**L1PL**	0.605	0.061	-0.042
**Acetoacetic acid**	0.668	0.000	0.260				
**Glucose**	0.666	0.001	-0.098				
**HDTG**	0.664	0.000	-0.115				
**V4CH**	0.663	0.001	-0.331				
**V4PL**	0.662	0.001	-0.329				
**Glutamic acid**	0.655	0.008	0.110				
**V4TG**	0.655	0.001	-0.207				
**Histidine**	0.651	0.006	-0.063				
**H3TG**	0.650	0.002	-0.444				
**H4FC**	0.650	0.003	0.165				
**IDCH**	0.650	0.001	-0.156				
**V4FC**	0.649	0.008	-0.770				
**Acetone**	0.647	0.008	0.120				
**IDFC**	0.647	0.001	-0.322				
**L1PN**	0.643	0.007	-0.031				

**L1AB**	0.642	0.007	-0.063
**VLPN**	0.642	0.004	-0.077
**VLAB**	0.642	0.004	-0.189
**H4A1**	0.637	0.006	0.033
**H4PL**	0.634	0.012	0.038
**V5TG**	0.633	0.032	-0.199
**2HB**	0.621	0.005	0.170
**TPAB**	0.613	0.050	-0.027
**TBPN**	0.613	0.050	-0.017
**TPA2**	0.612	0.068	0.024

Supplementary Table 25 – NMR parameters alone have only AUC 0.6-0.7 for classifying clinical stage of ovarian cancer.

Name	AUC	T-tests	Log2 FC	Name	AUC	T-tests	Log2 FC
**LDTG**	0.726	0.000	-0.098	**Histidine**	0.630	0.026	-0.051
**V4TG**	0.696	0.000	-0.258	**5CH**	0.629	0.097	-99.000
**H2TG**	0.695	0.000	-0.714	**V2CH**	0.622	0.014	-0.276
**L4TG**	0.694	0.002	-0.378	**L1PN**	0.620	0.049	-0.022
**L2TG**	0.694	0.002	-0.252	**L1AB**	0.620	0.050	-0.046
**V4PL**	0.693	0.000	-0.385	**H3FC**	0.619	0.048	0.146
**L5TG**	0.690	0.001	-0.436	**VLPL**	0.619	0.013	-0.109
**HDTG**	0.688	0.000	-0.125	**VLFC**	0.618	0.014	-0.139
**IDAB**	0.685	0.000	-0.226	**V5FC**	0.617	0.048	0.843
**IDPN**	0.685	0.000	-0.087	**V2FC**	0.614	0.010	-99.000
**V4CH**	0.684	0.000	-0.356	**V2PL**	0.610	0.030	-0.276
**H1TG**	0.683	0.000	-0.321	**Glycerol**	0.610	0.036	0.121
**VLPN**	0.677	0.000	-0.106	**TPA2**	0.610	0.070	0.024
**VLAB**	0.677	0.000	-0.260	**V2TG**	0.609	0.034	-0.122
**H4CH**	0.670	0.001	0.067	**Glucose**	0.607	0.018	-0.069
**Formic acid**	0.669	0.002	0.048	**L3FC**	0.604	0.055	0.098
**5TG**	0.669	0.002	-0.286	**IDTG**	0.601	0.034	-0.165
**L3TG**	0.667	0.008	-0.195	**HDA2**	0.600	0.100	0.020
**Glutamic acid**	0.665	0.002	0.130	**L3CH**	0.600	0.474	0.041
**V4FC**	0.661	0.002	-0.888	**VLTG**	0.600	0.037	-0.061
**V3PL**	0.653	0.002	-0.398	**L2CH**	0.595	0.059	0.080
**H4FC**	0.652	0.014	0.135				
**H3TG**	0.650	0.001	-0.482				

**IDCH**	0.650	0.001	-0.154
**V3FC**	0.648	0.002	-1.924
**TPTG**	0.643	0.005	-0.054
**IDFC**	0.643	0.001	-0.317
**H4A2**	0.643	0.006	0.063
**V3CH**	0.641	0.013	-0.352
**H4PL**	0.638	0.013	0.037
**V3TG**	0.637	0.008	-0.170
**VLCH**	0.636	0.003	-0.136
**L6TG**	0.636	0.006	-0.150
**V5PL**	0.635	0.120	-0.856
**H4A1**	0.634	0.015	0.029

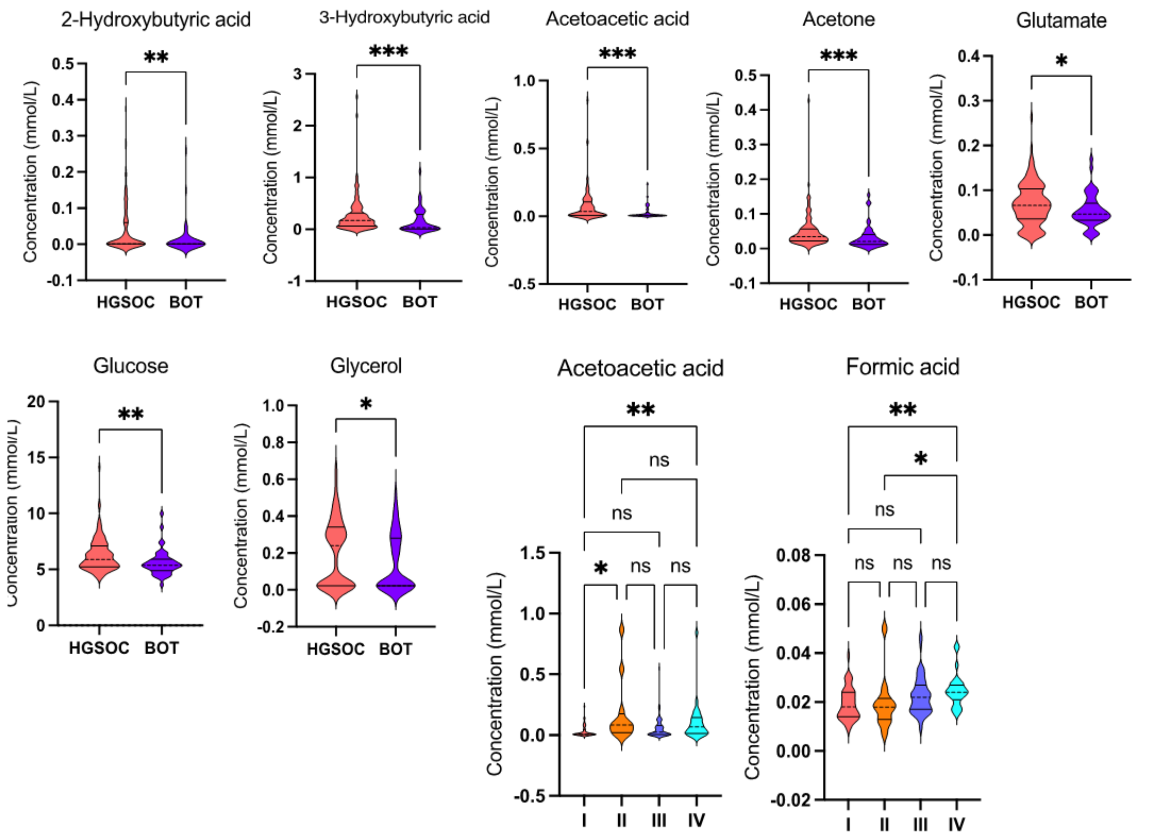

Supplementary Figure 1 – Altered metabolites in borderline ovarian tumor and high-grade serous ovarian cancer clinical stages I-IV.
Violin plots showing significant changes in metabolites in high-grade serous ovarian cancer and over the clinical stages by comparative statistics (*<0.05, ** < 0.01, ***< 0.001).

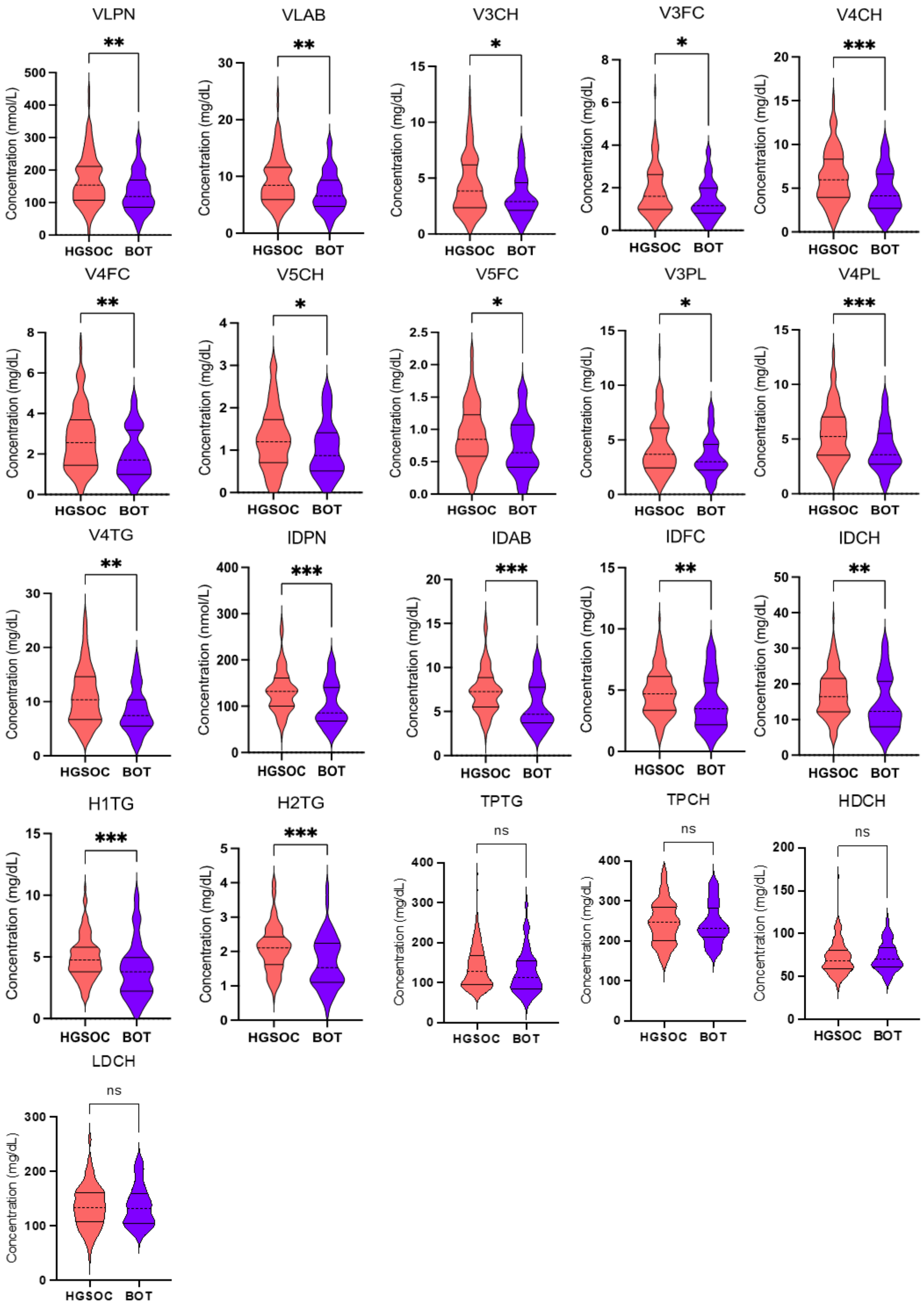

Supplementary Figure 2 – Levels of lipoproteins in borderline ovarian tumor and high-grade serous ovarian cancer serum samples.

Violin plots showing significant changes in lipoproteins in high-grade serous ovarian cancer by comparative statistics (ns = non-significance, *<0.05, ** < 0.01, ***< 0.001).

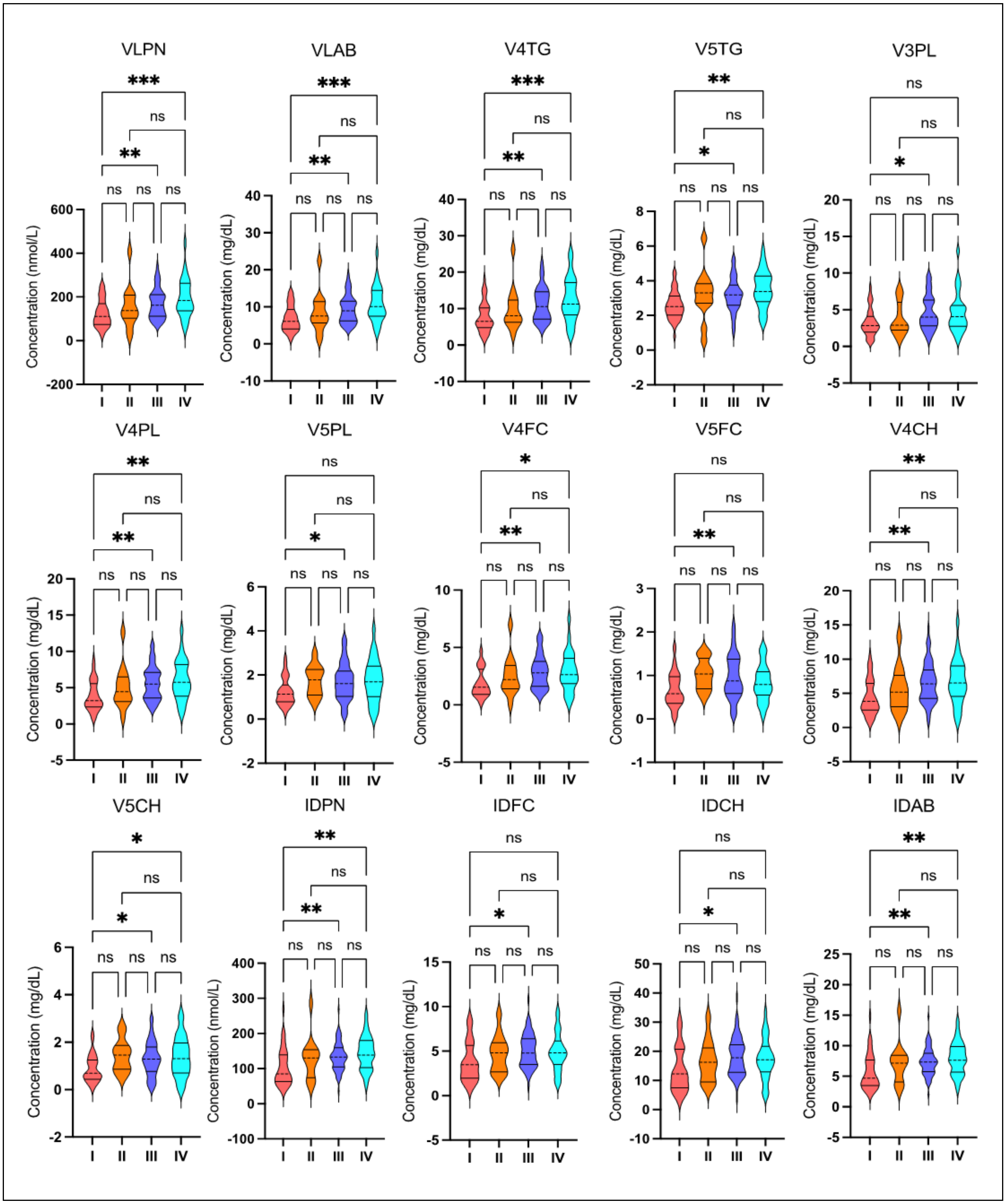
VLPN
***
ns
**
ns ns ns
Concentration (nmol/L)
600 400 200 0 -200
I II III IV
VLAB
***
ns
**
ns ns ns
Concentration (mg/dL)
40 30 20 10 0 -10
I II III IV
V4TG
***
ns
**
ns ns ns
Concentration (mg/dL)
40 30 20 10 0 -10
I II III IV
V5TG
**
ns
*
ns ns ns
Concentration (mg/dL)
8 6 4 2 0 -2
I II III IV
V3PL
ns
ns
*
ns ns ns
Concentration (mg/dL)
20 15 10 5 0 -5
I II III IV
V4PL
**
ns
**
ns ns ns
Concentration (mg/dL)
20 15 10 5 0 -5
I II III IV
V5PL
ns
ns
*
ns ns ns
Concentration (mg/dL)
6 4 2 0 -2
I II III IV
V4FC
*
ns
**
ns ns ns
Concentration (mg/dL)
10 5 0 -5
I II III IV
V5FC
ns
ns
**
ns ns ns
Concentration (mg/dL)
3 2 1 0 -1
I II III IV
V4CH
**
ns
**
ns ns ns
Concentration (mg/dL)
20 15 10 5 0 -5
I II III IV
V5CH
*
ns
*
ns ns ns
Concentration (mg/dL)
6 4 2 0 -2
I II III IV
IDPN
**
ns
**
ns ns ns
Concentration (nmol/L)
400 300 200 100 0 -100
I II III IV
IDFC
ns
ns
*
ns ns ns
Concentration (mg/dL)
15 10 5 0 -5
I II III IV
IDCH
ns
ns
*
ns ns ns
Concentration (mg/dL)
50 40 30 20 10 0 -10
I II III IV
IDAB
**
ns
**
ns ns ns
Concentration (mg/dL)
25 20 15 10 5 0 -5
I II III IV

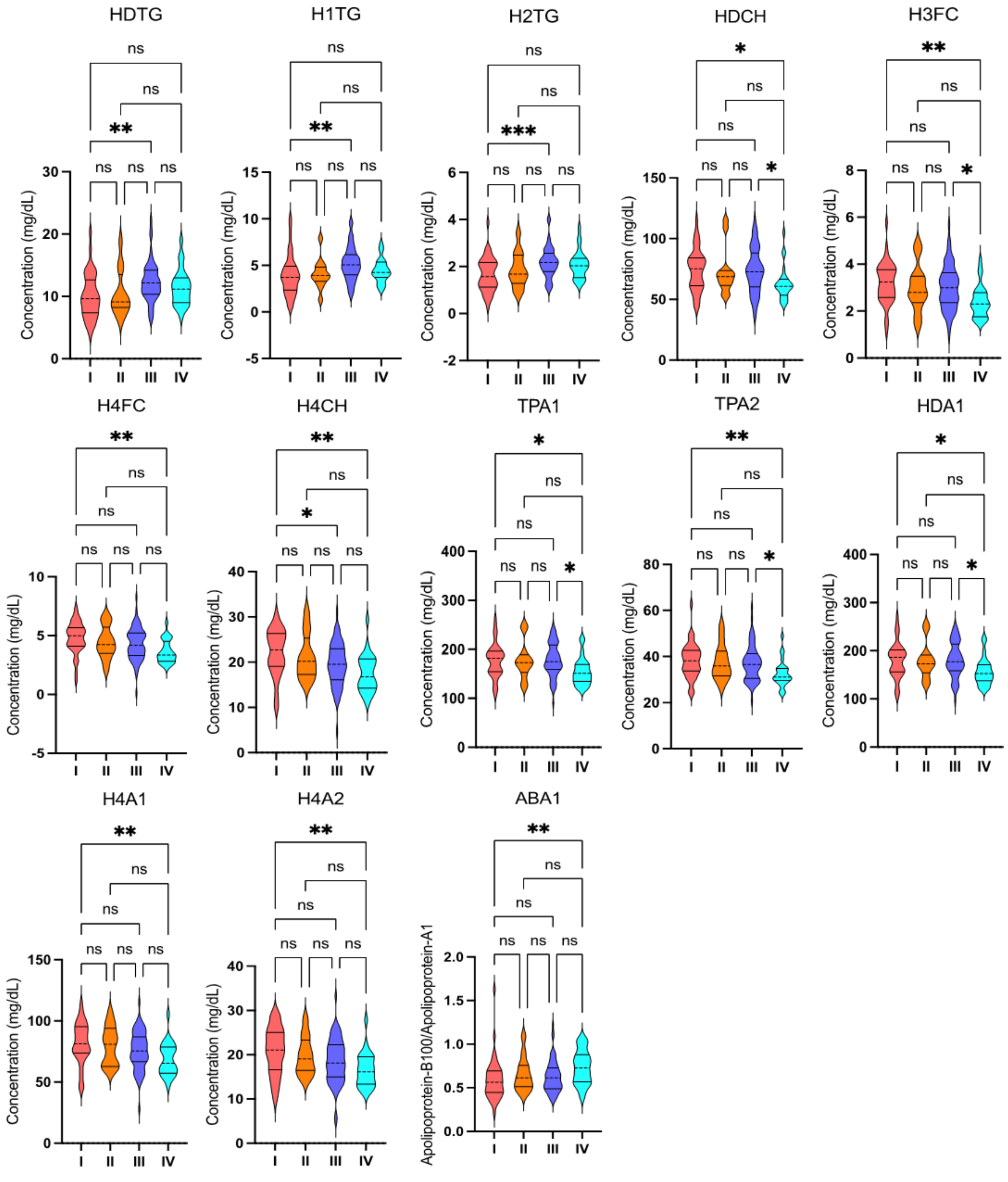

Supplementary Figure 3 – Altered of lipoproteins in borderline ovarian tumor and high-grade serous ovarian cancer clinical stages I-IV.

Violin plots showing significant changes in lipoproteins over the clinical stages by comparative statistics (ns = non-significance, *<0.05, ** < 0.01, ***< 0.001).

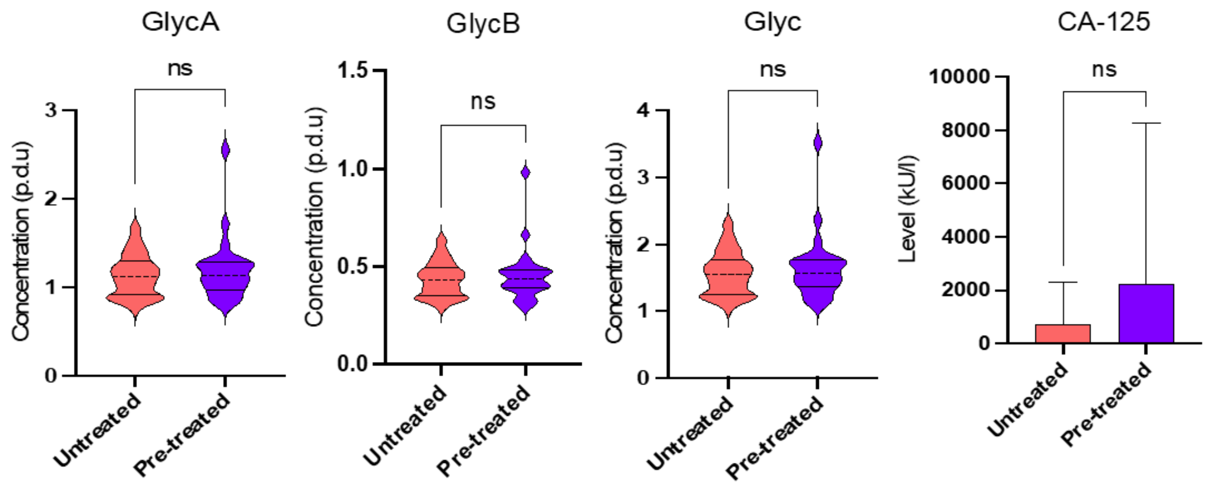

Supplementary Figure 4 – Levels of glycoprotein inflammation and cancer antigen-125 markers in untreated and pre-treated patients.

Violin and box plots showing non-significant changes in glycoprotein inflammation and cancer antigen-125 markers between the treatment status by comparative statistics (ns = non-significance).

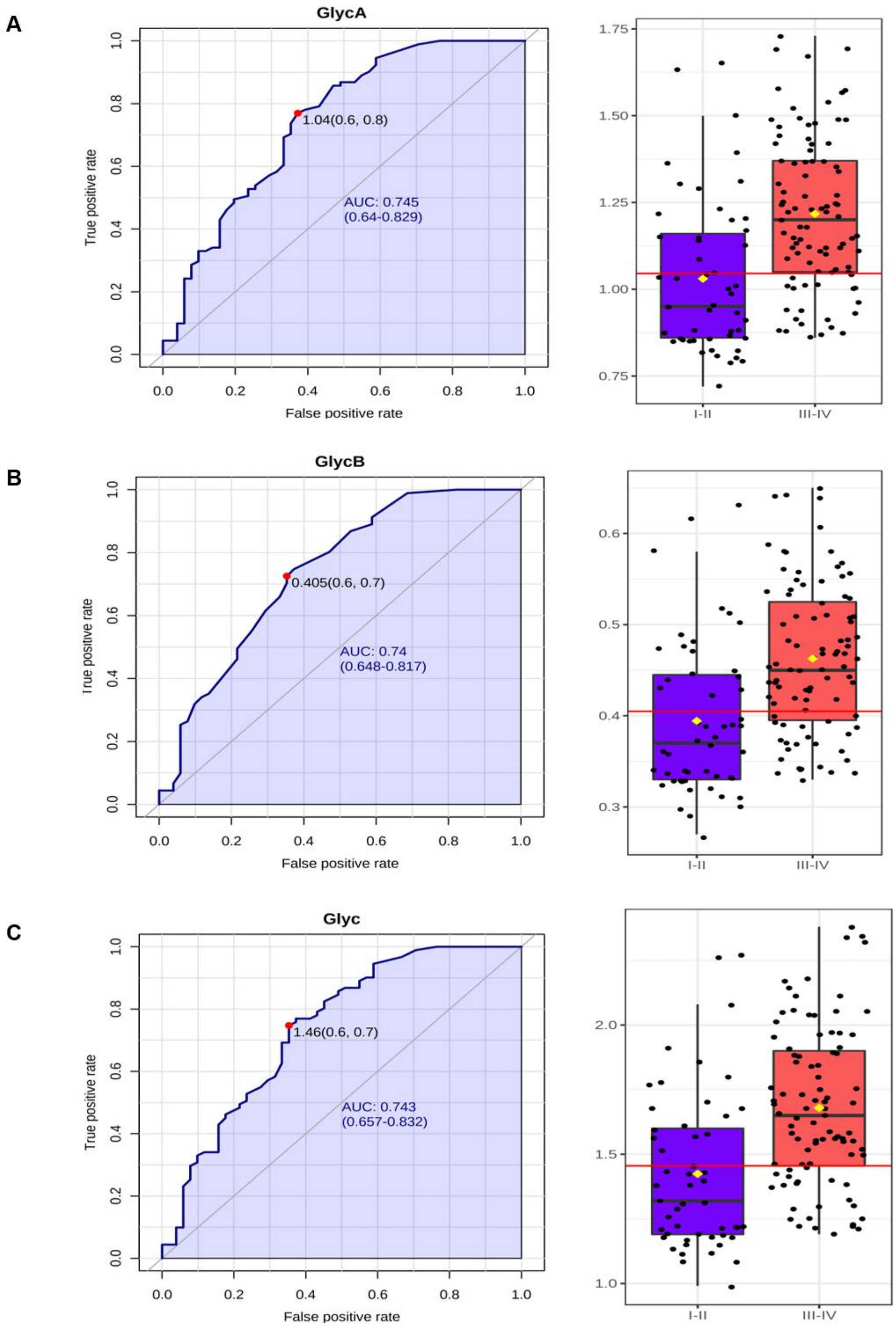

Supplementary Figure 5 – Glycoprotein inflammation markers for ovarian cancer diagnosis and prognosis.

The optimal cut-off was based on the closest to the top left corner principle and is indicated by the red dot in all the ROC curves. Black dots and yellow diamond represent GlycA, GlycB and Glyc and mean concentration of GlycA, GlycB and Glyc, respectively.

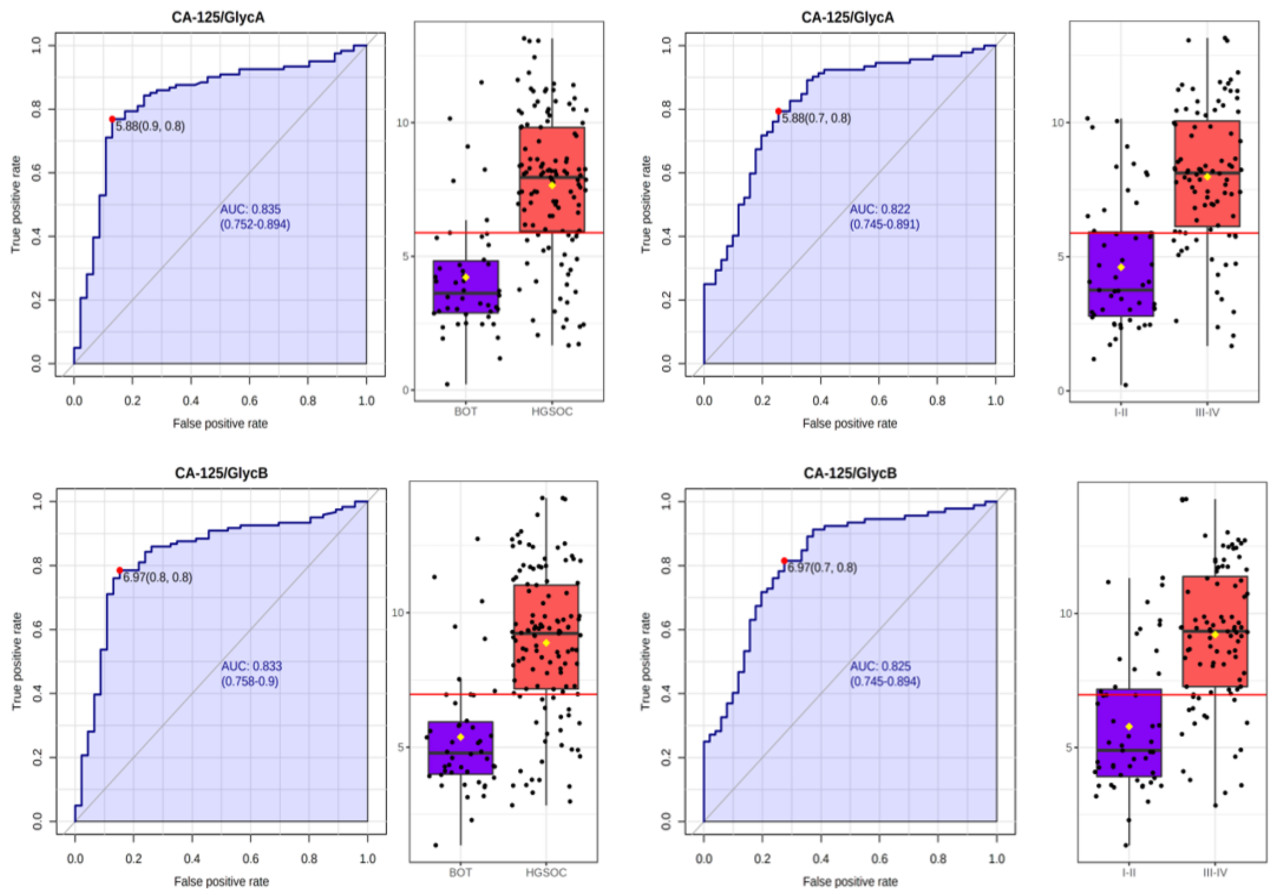

Supplementary Figure 6 – CA125/glycoprotein inflammation markers for ovarian cancer diagnosis and prognosis.

The optimal cut-off was based on the closest to the top left corner principle and is indicated by the red dot in all the ROC curves. Black dots and yellow diamond represent each ratio and mean concentration of each ratio, respectively.

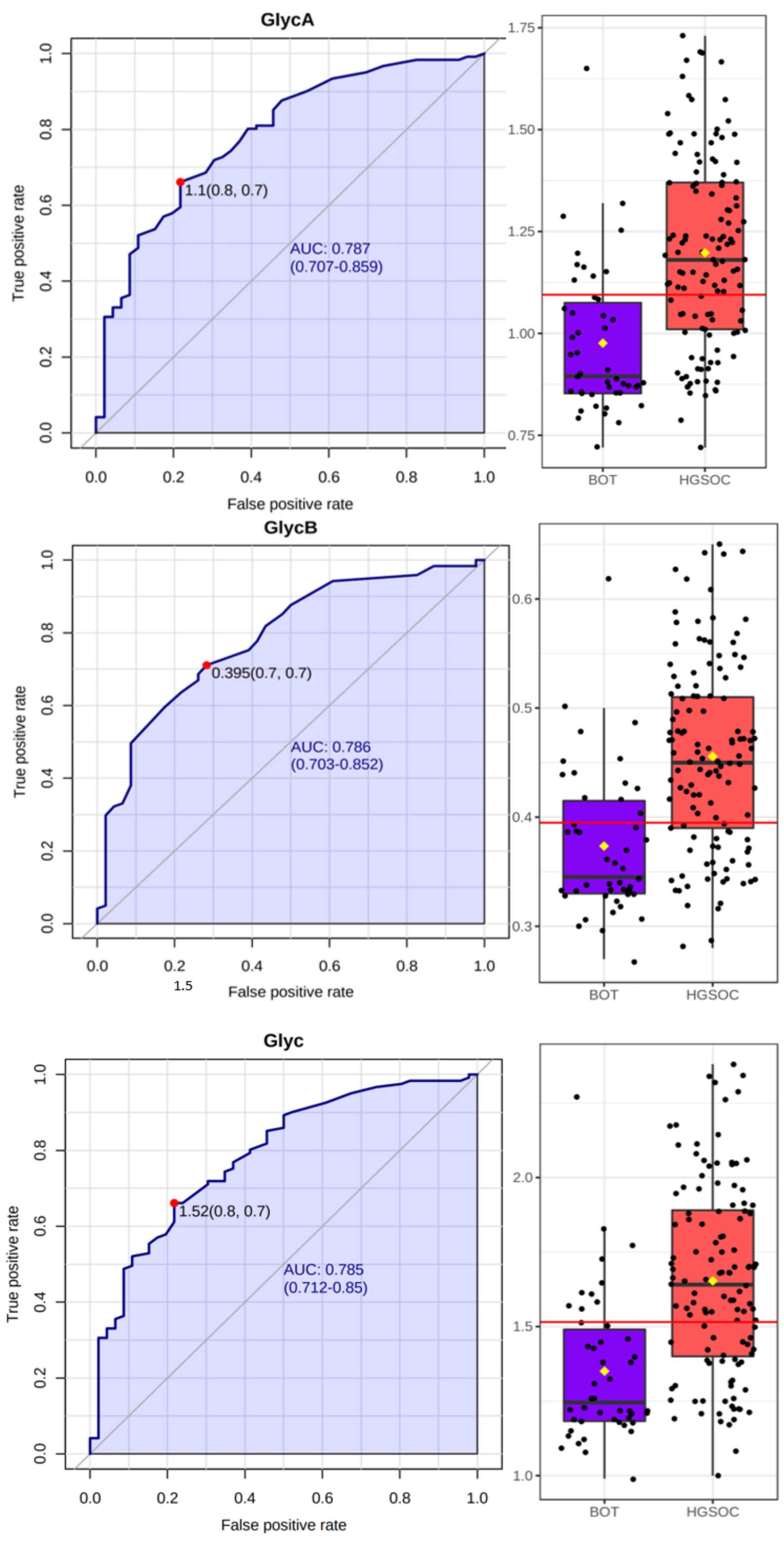

Supplementary Figure 7 – Glycoprotein inflammation markers for ovarian cancer diagnosis and prognosis.

The optimal cut-off was based on the closest to the top left corner principle and is indicated by the red dot in all the ROC curves. Black dots and yellow diamond represent GlycA, GlycB and Glyc and mean concentration of GlycA, GlycB and Glyc, respectively.

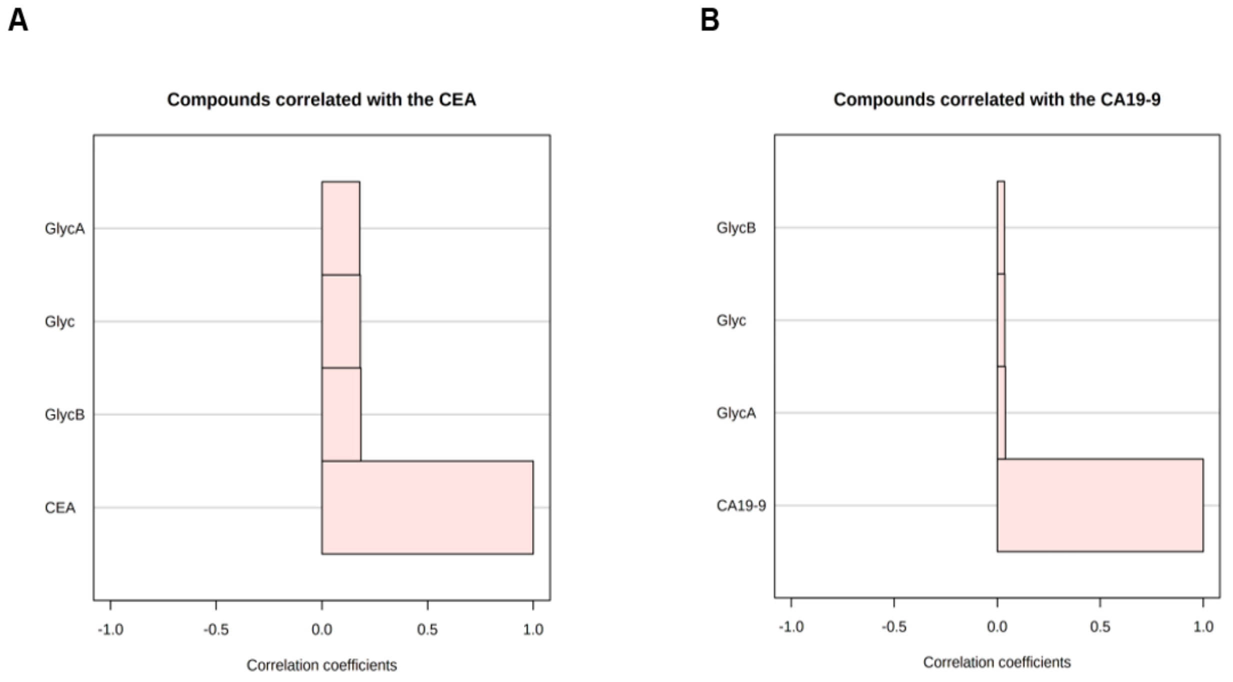

Supplementary Figure 8 – correlation of glycoprotein inflammation with carcinoembryonic antigen and cancer antigen 19-9.
A & B) Correlation of glycoprotein inflammation markers with carcinoembryonic antigen and cancer 19-9 markers showing weak positive correlation.

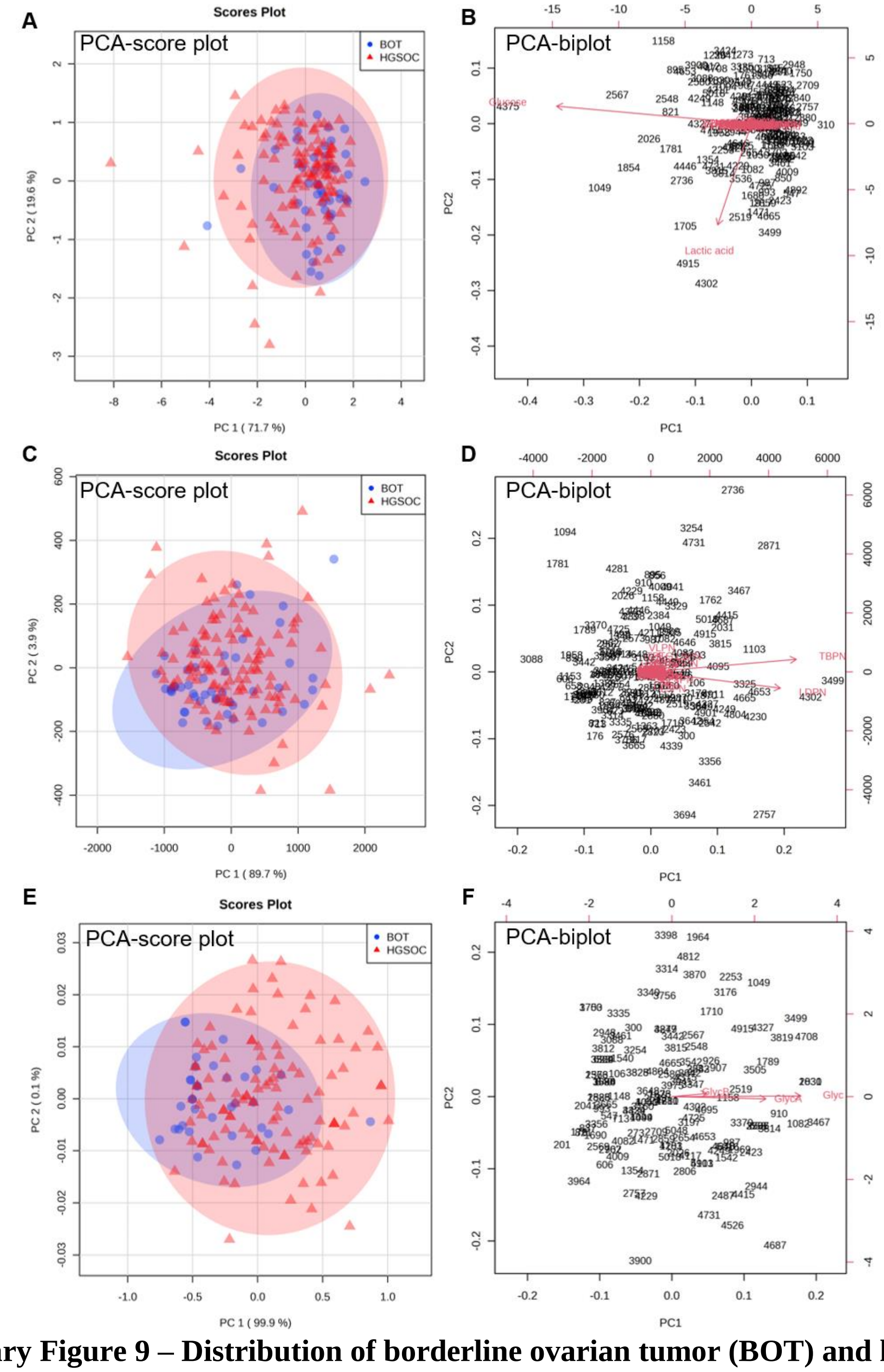

Supplementary Figure 9 – Distribution of borderline ovarian tumor (BOT) and high-grade serous ovarian cancer (HGSOC) with clinical stages I-IV.

A, C and E) PCA-score plots; (A) metabolites, (B) lipoproteins and (E) Inflammation markers. B, D and F) PCA-biplots; (B) metabolites driving separation are glucose and lactic acid, while (D) lipoproteins (TBPN and LDPN), and (F) inflammation markers (GlycA, GlycB and Glyc).

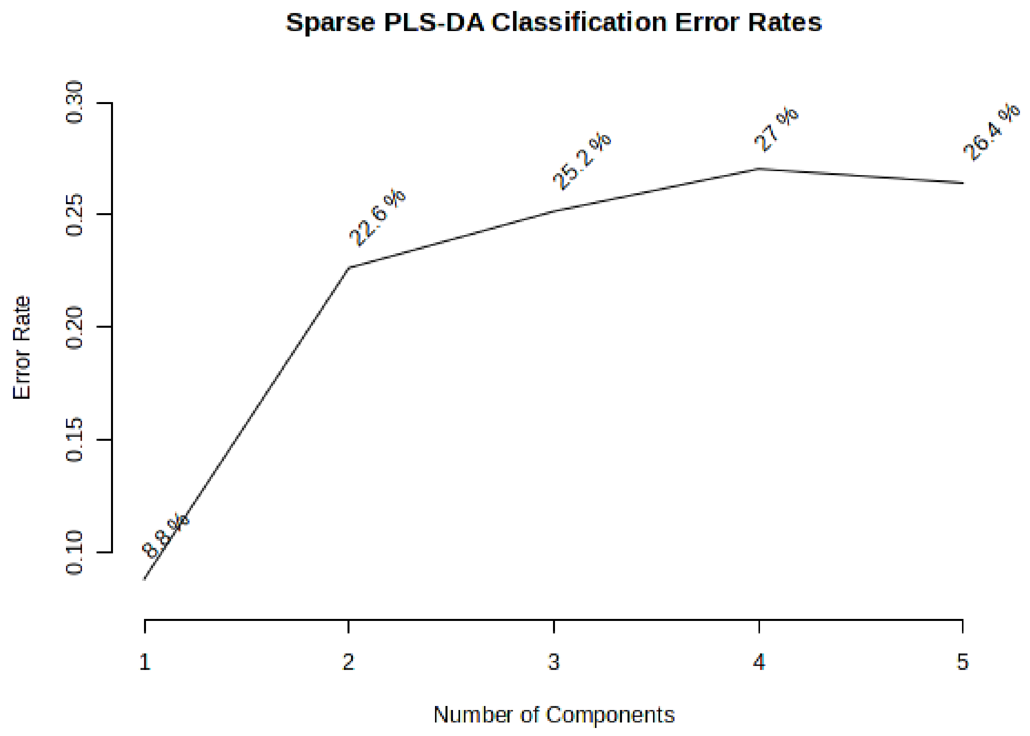

Supplementary Figure 10 – Leave one out cross-validation for sPLSDA model of different glycoprotein classes.

sPLSDA model that is based on different glycoprotein classes was evaluated by leave on out cross-validation. Component has the lowest error rate (8.8%) among the components. In other words, the model with component 1 provide good classification based on different glycoprotein classes.
